# Revisiting the Robin–Day Classification through Switchable Electronic States in Multimetallic Vanadium Oxides

**DOI:** 10.1021/jacs.6c00579

**Published:** 2026-04-14

**Authors:** Nghia Le, Pere Miró

**Affiliations:** Department of Chemistry, 4083 University of Iowa, Iowa City, Iowa 52242, United States

## Abstract

Polyoxovanadate–alkoxide clusters are redox-active molecular oxides offering profound electronic tunability. The oxygen-deficient species [(V_6_O_5_)­(μ_6_-O)­(μ_2_-OCH_3_)_12_] is an ideal platform for probing the multisite localization and delocalization of redox states. Here, we introduce the redox topology modulation which is governed by the position of the central μ_6_-O oxygen and ligand coordination at the oxygen-deficient site as the mechanism controlling the stability of different electromers. The noncoordinated cluster exhibits a localized, Robin–Day class I/II hybrid ground state, featuring a V­(III) center at the vacancy defect. We demonstrate computationally that ligand-field tuning inverts this behavior; coordination of a strong donor destabilizes the localized topology, stabilizing an electromer with all V­(IV) topology as the new ground state. Time-dependent density functional theory calculations show that photoexcitation of species where centers are V­(IV) triggers photoinduced intervalence charge transfer regenerating a valence-trapped class II excited state. This work establishes the redox topology modulation as a rational design principle for molecular switches, where the fundamental electronic topology can be toggled by chemical stimulus and/or by light. Furthermore, our results suggest that the Robin–Day classification should be revised and extended for multicenter systems, where valence behavior is better understood as excitation-specific rather than molecular-specific.

## Graphical abstract

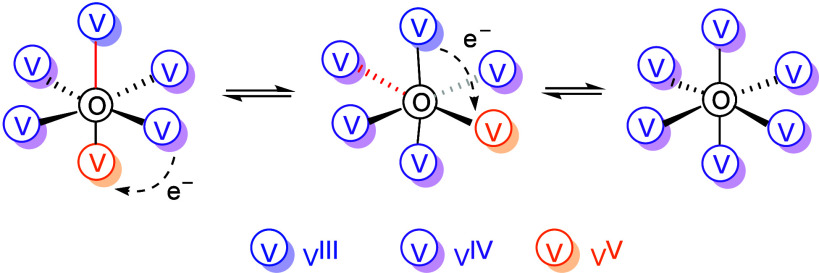

Polyoxovanadate–alkoxide (POV) clusters are a diverse class of molecular metal-oxides well-known for their rich redox chemistry.^1,2^ Their ability to host multiple redox-active vanadium centers in close proximity, combined with the tunability of their surface alkoxide ligands, makes them ideal systems for probing the fundamental principles of mixed-valence chemistry.^3,4^ The electronic structure of such compounds has traditionally been understood through the Robin-Day classification, which distinguishes between fully localized, noninteracting (Class I), valence-trapped, but electronically coupled (Class II), and fully delocalized (Class III) regimes.^5,6^ Understanding the structural drivers of transitions between these regimes is essential for designing multimetallic molecular materials with well-defined redox behaviors.

In the POV family, the Lindqvist-type species [(V^V^_6–*n*_V^IV^_*n*_O_6_)­(μ_6_-O)­(μ_2_-OCH_3_)_12_]^4–*n*^ (**1**) are a particularly robust scaffold and they have been identified as a canonical class II mixed-valence system (Figure 1, left).^7^ However, the removal of a terminal oxo moiety to form its oxygen-deficient congener, [(V^III^)­(V^V^_5–*n*_V^IV^_*n*_O_5_)­(μ_6_-O)­(μ_2_-OCH_3_)_12_]^4–*n*^ (**2**) (Figure 1, right), raises a critical question regarding this classification.^8^ The vanadium center at the oxo defect site exhibits unusual behavior as it remains V­(III) upon cluster oxidation suggesting this site is electronically isolated, indicating a shift from the expected class II toward a hybrid class I/II regime.^9^

**Figure 1:**
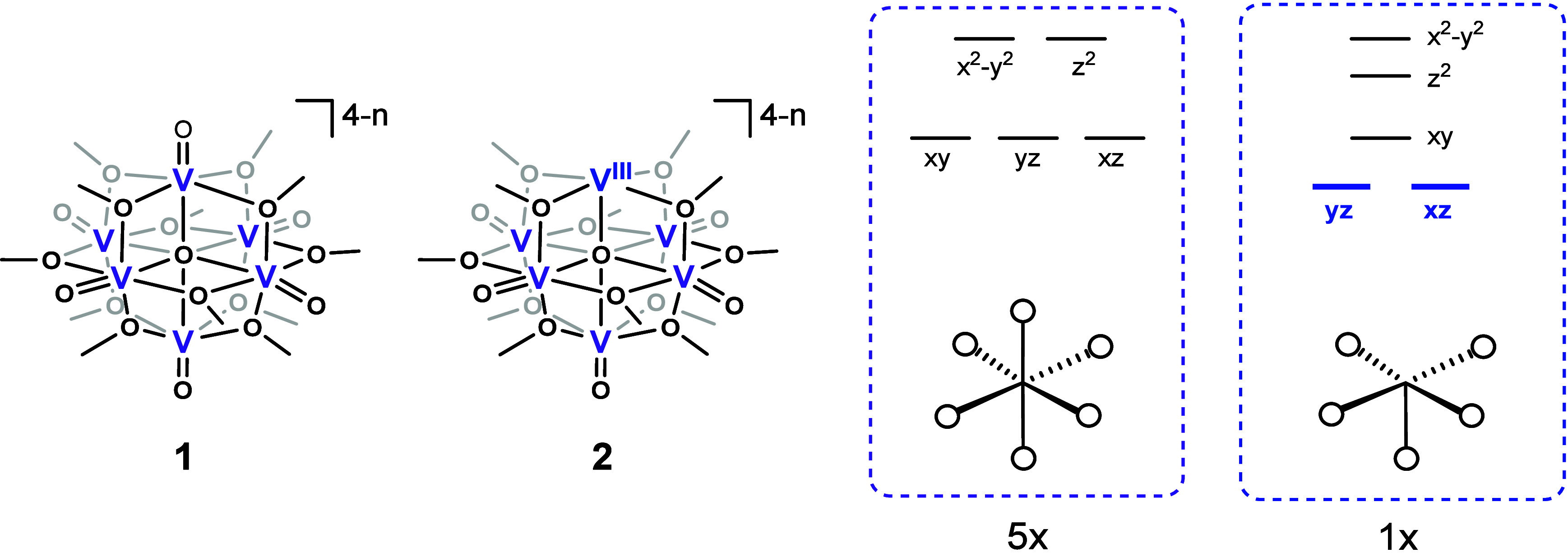
Structures of the pristine polyoxovanadate [(V^V^_6–*n*_V^IV^_*n*_O_6_)­(μ_6_-O)­(μ_2_-OCH_3_)_12_]^4–*n*^ (**1**), its O-deficient congener [(V^III^)­(V^V^_5–*n*_V^IV^_*n*_O_5_)­(μ_6_-O)­(μ_2_-OCH_3_)_12_]^4–*n*^ (**2**), and the local vanadium ligand field diagrams for a *O*_*h*_ and defective vanadium site.

This apparent valence-trapping at the defect site can be qualitatively rationalized by its local ligand field. Removal of the strong *σ*- and *π*-donating oxo ligand lowers the frontier *d*-orbital energies, particularly those with *z*-character (*d*_*xz*_ and *d*_*yz*_), stabilizing a static V­(III) (*d* ^*2*^) center (Figure 1). While this local picture explains the observed retention of V­(III) upon oxidation, it does not address the interactions among the remaining five vanadium centers or how the cluster’s global electronic structure responds to redox events. A full quantum-mechanical description is therefore required to capture the complete redox topology of **2** as the total charge of the complex is varied. This is particularly important since oxygen-deficient clusters act as molecular models of local metal–oxo defect chemistry, conceptually bridging coordination chemistry and oxide surfaces, as first highlighted by Pope and Müller over three decades ago.^10^ With recent synthetic advances making these defect sites readily accessible,^11–13^ the need for a predictive theoretical framework to model their electronic behavior has become essential.

We began our investigation of **2** with the neutral oxygen-deficient species, which contains six *d*-electrons among its six vanadium centers. DFT calculations identify three distinct electromers^14^ (Figure 2 top), which are redox topologies with similar geometries but different electron distributions. The thermodynamic ground state is a localized *trans* isomer that maximizes the spatial separation between the V­(III) *d* ^2^ defect site and a V­(V) *d* ^0^ center. A related *cis* topology, in which the V­(V) center is positioned in the equatorial plane, lies only 0.5 kcal/mol higher in energy. In sharp contrast, a more uniform electron distribution with an *all-IV* state, which is significantly less stable given that it requires the V­(III) defect to oxidize, residing 7.5 kcal/mol above the ground state (see Figure S5 in the Supporting Information (SI) for spin densities). This large thermodynamic penalty for delocalization confirms that this state is less accessible. The ground-state dynamics are therefore restricted to interconversion between the localized *cis* and *trans* isomers. Since the vanadium center at the defect site remains V­(III) in both stable topologies, it behaves as a static, class I center. Overall, after removing oxo moiety, the cluster turns from pure class II to the hybrid class I/II. Multireference calculations agree with the DFT results, confirming that all spin states are within a few kcal/mol and that can be assigned to the same redox topologies previously described. The *trans* redox topology is always favored over the *cis* and the all V­(IV) species at the CASPT2 level of theory, specifically by 4.5 and 29.6 kcal/mol respectively (see Section S1.3 in the SI for details).

**Figure 2:**
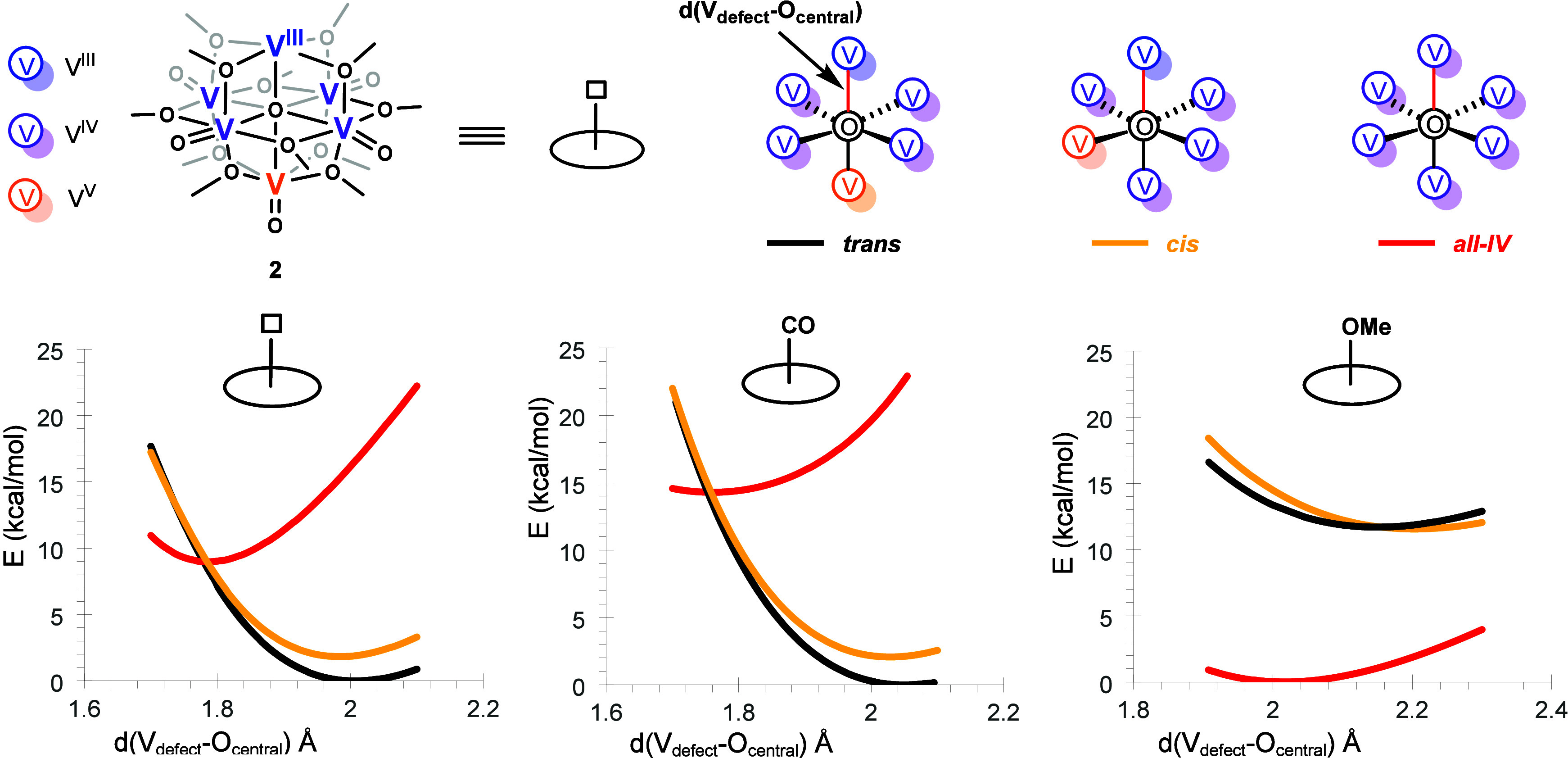
Schematic representation of different redox topologies for the oxygen-deficient species (top). Potential energy surfaces respect to the V_defect_–O_central_ moiety with different ligands at the defect site (bottom).

These relative energies correspond to adiabatic minima, not vertical excitations. Although the isomers share similar geometries, their key structural difference lies in the position of the central μ_6_-O atom. This deviation is electrostatically driven since the μ_6_-O^2–^ atom shifts to minimize repulsion, moving away from the electron-rich V­(III) center and toward the electrophilic V­(V) center. Related redox-active behavior of oxygen ligands has been increasingly recognized in transition-metal chemistry.^15–17^ Due to the rigidity of the Lindqvist framework, this motion is highly coupled. Elongation of the V­(III)–O_central_ bond induces a concurrent shortening of the V–O_central_ bond trans to it and vice versa. This direct structural correlation dictates the localization of redox states and constitutes the physical origin of redox topology modulation (RTM).

To visualize the RTM, the ground-state diabatic potential energy surfaces were mapped along the V_defect_–O_central_ coordinate (Figure 2 bottom). This projection reveals two distinct electronic manifolds. The low-energy manifold is comprised of the two localized isomers: the thermodynamic ground state (*trans*, black curve) and the low-lying *cis* isomer (orange curve). Critically, their potential wells exhibit significant fractional overlap, with minima at 2.01 Å and 1.98 Å, respectively. This is consistent with the small energy gap separating them. In contrast, the *all-IV* state (red curve) constitutes a distinct, high-energy surface with its minimum at a much shorter distance of 1.74 Å. The diabatic crossing between the localized manifold and this *all-IV* state occurs at ∼1.8 Å. This corresponds to a diabatic thermal barrier of 8.4 kcal/mol from the *trans* isomer minimum energy structure and represents the reorganization energy for this process before electronic coupling is considered. While this is a simplification of a complex multidimensional potential energy surface, it effectively illustrates how the energetic landscape and redox topologies are governed by the position of the central μ_6_-O atom.

Given the small *cis*–*trans* energy gap, we posited that RTM could be modulated through coordination at the open defect site. We selected −CO (π-acceptor) and −OCH_3_ (strong σ-donor) ligands to probe this effect, and the resulting surfaces are shown in Figure 2 bottom. Coordination of the *π-*accepting −CO ligand further stabilizes the V­(III)/V­(V) states, widening the energy gap with the *all-IV* state. In contrast, methoxide dramatically destabilizes the localized *cis* and *trans* isomers, causing the *all-IV* surface to drop in energy and become the new thermodynamic minimum. This inversion arises from repulsive interactions between the electron-rich methoxide ligand and the V­(III) *d* ^*2*^ center, rendering the defect more easily oxidized and eliminating its class I behavior. The six valence electrons therefore redistribute more evenly across the cluster. This electronic switch correlates with structural response: the V_defect_–O_central_ bond elongates from 1.79 Å (noncoordinated, **2-** ***trans***) to 1.82 Å with the carbon monoxide ligand and to 2.02 Å with the methoxy ligand. Recent experimental work by Matson and co-workers on a TMSO-bound cluster shows exactly this delocalized *all-IV* configuration,^18^ consistent with our prediction for methoxide coordination. This confirms that the electronic ground state of POV clusters can be tuned from a more localized to a more delocalized *all-IV* topology via ligand-field control at the defect site (see Figures S5 and S6 in the SI for relative Gibbs free energies). A systematic ligand analysis shows that strong σ-donor character and anionic ligands stabilize the *all*-IV state (see Section S1.5 in the SI).

Notably, while the *all-IV* state features an even distribution of *d*-electrons among the six vanadium centers, it does not conform to the conventional Robin–Day class III description. Traditional mixed-valence theory, as developed by Hush and Marcus, builds on a two-site donor–acceptor model described by a double-well potential, where strong electronic coupling collapses the barrier to yield a single delocalized minimum.^19,20^ In contrast, the present cluster undergoes intervalence charge transfer (IVCT) across multiple subsets of vanadium centers. The resulting excited states fall into a regime of fractional delocalization that is poorly described by the canonical class III potential,^21^ necessitating a state-specific treatment that explicitly accounts for multicenter IVCT.

To rationalize the complex excited-state dynamics, we introduce a simplified three-site model that captures the two IVCT pathways identified in our calculation: delocalization-driven redistribution (DDR) and charge transfer-driven redistribution (CTDR) (for assignment details, see Section S1.4 in the SI). These mechanisms differ in whether charge transfer is intrinsic to the vertical excitation or arises only during relaxation In the DDR pathway (Figure 3 top), the vertical excitation populates a delocalized 2-electron/3-center manifold rather than a defined donor–acceptor state. Relaxation within this surface redistributes the V­(IV) character among the participating centers and can even restore the initial redox topology. In the CTDR pathway (Figure 3 bottom), the excitation directly transfers electron density from a multicenter donor manifold to a specific acceptor, formally reducing V­(V) to V­(IV). The resulting hole is initially distributed over several donor sites but becomes localized at one site as the system relaxes, generating a new V­(V) center. This pathway imposes a directional redox change and does not return to the starting configuration.

**Figure 3:**
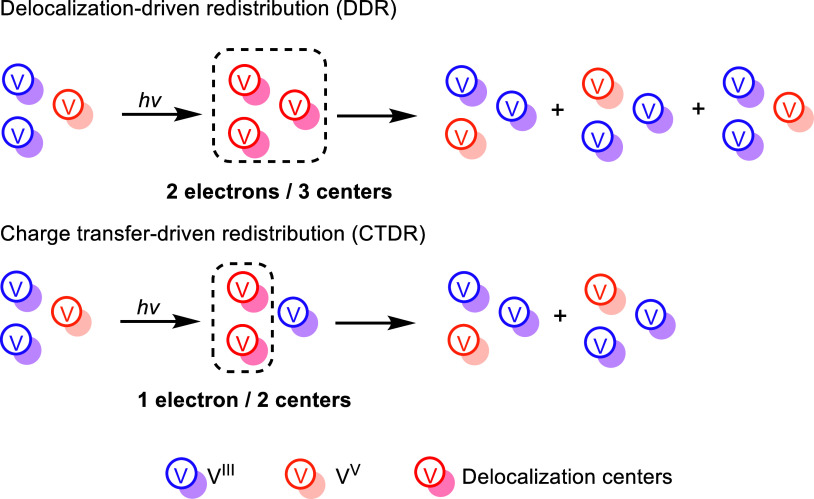
Delocalization-driven redistribution (DDR) mechanism (top) and charge transfer-driven redistribution (CTDR) mechanism (bottom).

Relative to conventional two-site models, charge-transfer character is expected to become increasingly delocalized near crossings of the diabatic surfaces.^22^ The observation of a DDR pathway directly at the ground-state minimum therefore indicates strong electronic mixing among adjacent vanadium atoms. This mixing is amplified by the cluster symmetry: the four equatorial vanadium centers are chemically equivalent, enabling redistribution channels characteristic of the highly symmetric class [II + II] regime.^23^

Both mechanisms appear across different vanadium sites depending on the excitation energy. For example, the near-infrared transitions at λ = 861.8 nm (**2-** ***cis***) and 762.9 nm (**2-** ***trans***) interconvert the two topologies through DDR and CTDR, respectively (Figure 4 left). Although IVCT bands in the 700–1000 nm region are typically assigned to V­(IV)→V­(V) transitions,^13^ our analysis shows that multiple V­(IV) centers can participate in a single IVCT event. Furthermore, the coordination of −OCH_3_ stabilizes an all-V­(IV) ground state and reshaping the IVCT character. In this case, the lowest-energy transition occurs in the visible range at λ = 667.5 nm and corresponds to CTDR in which the electron density is transfer from a five-center donor manifold into the defect-site vanadium, formally generating a V­(III) center and accessing excited states of both *cis* and *trans* topologies (Figure 4 right). This assignment is supported by comparison to the −OTMS analogue, whose experimental IVCT band near 620 nm is reproduced by our model at 658.9 nm (Figure S4).^18^ The resulting excited-state reorganization resembles photoinduced IVCT in two-site systems, where a delocalized ground state becomes localized upon photoexcitation.^24^

**Figure 4:**
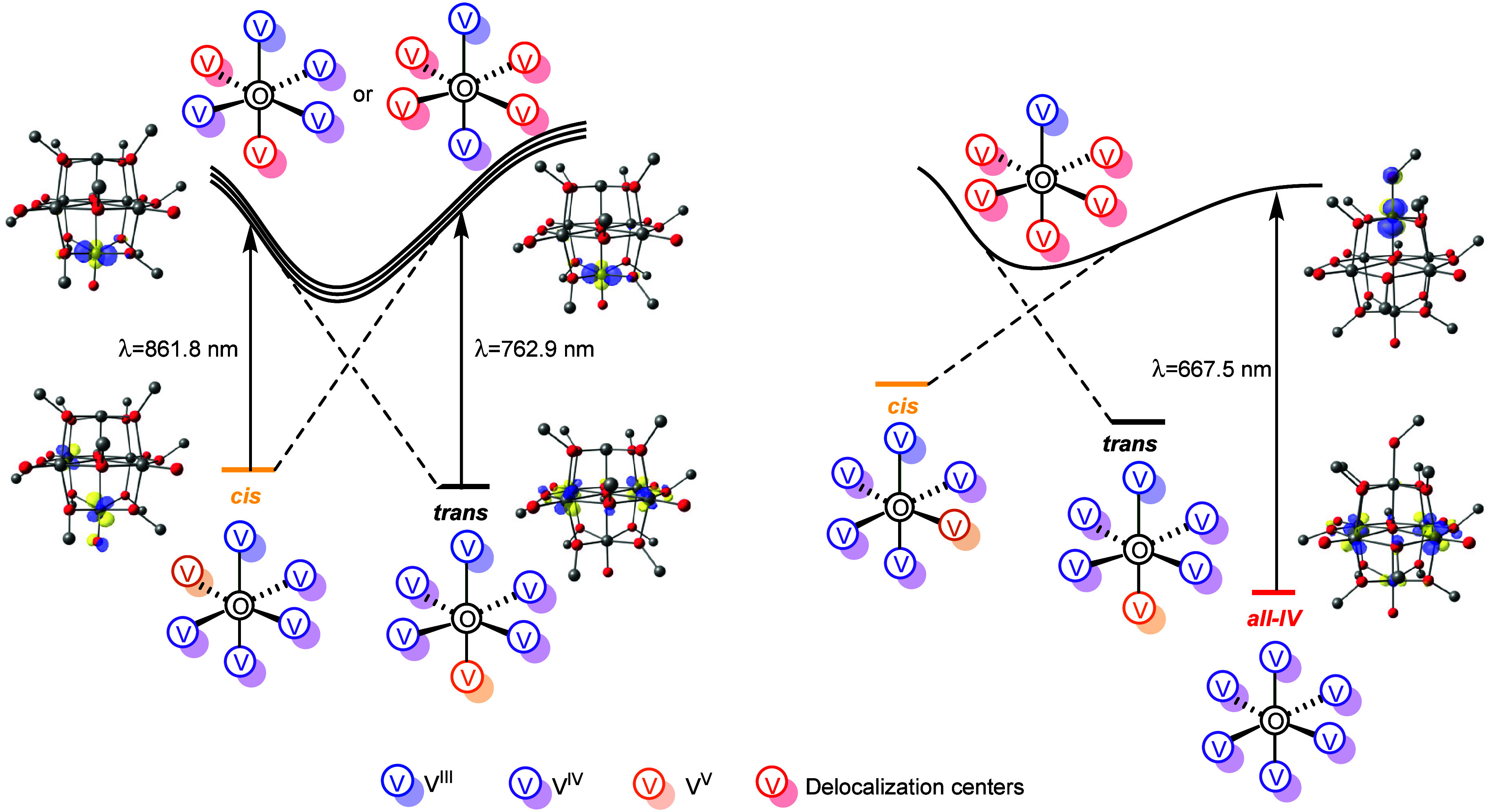
Selected transitions and corresponding natural transition orbitals for the interconversion between **2-*cis*** and **2-*trans*** redox topologies (left) and the photo induced IVCT from the all-V­(IV) state to the *cis* and *trans* topologies of the methoxy-bound complex (right).

Within a single molecule, different excitations engage distinct subsets of vanadium centers, and each transition can proceed through either the DDR or CTDR mechanism, generating its own transient donor–acceptor pattern (see Tables S1–S4 in the SI for full assignments). Because these IVCT pathways frequently involve more than two metal centers, they extend beyond the two-site models underlying the Robin–Day framework. Consequently, IVCT in these systems is inherently excitation-specific: no single Robin–Day class can describe the entire molecule, and each transition exhibits its own mixed-valence character defined by the associated redistribution of electron density, an assignment that requires a computational analysis.

In summary, oxygen-deficient vanadate clusters exhibit a switchable electronic structure in which the balance between localized and delocalized mixed-valence configurations is strongly tuned by ligand fields. An anionic strong donor ligand such as methoxide stabilizes an *all-IV* thermal ground state relative to the *cis* and *trans* topologies, which can be transiently accessed upon photoexcitation. Individual excitations engage different subsets of vanadium centers and activate distinct multimetal redistribution pathways described by the DDR and CTDR mechanisms, providing a framework for describing IVCT processes in multicenter mixed-valence compounds.

## Supplementary Material



## Data Availability

All optimized cartesian coordinates (XYZ), along with inputs and outputs files for all calculations, can be found in FigShare (https://figshare.com/s/eecc1cdd18e9ca2205de).

## Notes

Computational details, electronic absorption spectra, natural transition orbitals, spin densities, active space natural orbitals, and CASSCF/CASPT2 energies (PDF)
