# Supplementary material for: Revisiting the Robin–Day Classification through Switchable Electronic States in Multimetallic Vanadium Oxides
Source: J Am Chem Soc. 2026 Apr 14;148(16):16583–7. doi: 10.1021/jacs.6c00579 (PMC13133899; doi:10.1021/jacs.6c00579)
Supplement: Supplementary file 1 [file ja6c00579_si_001.pdf]

Supporting Information

Revisiting the Robin–Day Classification through Switchable Electronic States in Multi-metallic Vanadium Oxides

Nghia Le and Pere Miró*

pere-miro@uiowa.edu

Department of Chemistry, University of Iowa, Iowa City, Iowa 52242, USA

Table of Contents

S1.1 Density Functional Theory Calculations 5

S1.2 Verification of the Computational Approach 5

S1.3 Complete Active Space Calculations...5

S1.4 Influence of Ligand and Charge on the Stability of Redox Topologies.........6

S1.5 Assignment of intervalence charge transfer..7

S.2 Supplementary Figures 9

Figure S1. Simulated electronic absorption spectrum of **$[(V_6O_5)(\mu_6\text{-}O)(\mu_2\text{-}OCH_3)_{12}]^-$** at SMD(THF)-PBE0/def2-TZVP-D4//BS1 level theory, with selected NTOs for key transitions..9

Figure S2. Simulated electronic absorption spectrum of compound **2-*cis***.................10

Figure S3. Simulated electronic absorption spectrum of compound **2-*trans***. 11

Figure S4. Simulated electronic absorption spectrum of compound **2-OTMS-*All-IV***. 12

Figure S5. Schematic representation of different redox topologies of the oxygen-deficient **2** species (top) and DFT spin density (bottom)...13

Figure S6. Different redox topologies of **2-CO** (top) and **2-OMe** (bottom) species 14

Figure S7. Relative stabilities of trans, *cis*, and *all-IV* topologies influenced by selected neutral ligands 15

Figure S8. Relative stabilities of *cis*, *trans*, and *all-IV* topologies influenced by selected anionic ligands...16

Figure S9. Gibbs free energy (kcal/mol) comparison of redox topologies influenced by various ligands. 17

Figure S10. Active space natural orbitals (isovalue=0.04) with occupation numbers (in parenthesis) greater than 0.02 for the septet state of species **2-*trans*** with no ligand at CASSCF level of theory...18

Figure S11. Active space natural orbitals (isovalue=0.04) with occupation numbers (in parenthesis) greater than 0.02 for the quintet state of species **2-*trans*** with no ligand at CASSCF level of theory...19

Figure S12. Active space natural orbitals (isovalue=0.04) with occupation numbers (in parenthesis) greater than 0.02 for the triplet state of species **2-*trans*** with no ligand at CASSCF level of theory. 20

Figure S13. Active space natural orbitals (isovalue=0.04) with occupation numbers (in parenthesis) greater than 0.02 for the singlet state of species **2-*trans*** with no ligand at CASSCF level of theory...21

Figure S14. Active space natural orbitals (isovalue=0.04) with occupation numbers (in parenthesis) greater than 0.02 for the septet state of species ***2-all-IV*** with no ligand at CASSCF level of theory....22

Figure S15. Active space natural orbitals (isovalue=0.04) with occupation numbers (in parenthesis) greater than 0.02 for the quintet state of species ***2-all-IV*** with no ligand at CASSCF level of theory....23

Figure S16. Active space natural orbitals (isovalue=0.04) with occupation numbers (in parenthesis) greater than 0.02 for the triplet state of species ***2-all-IV*** with no ligand at CASSCF level of theory. 24

Figure S17. Active space natural orbitals (isovalue=0.04) with occupation numbers (in parenthesis) greater than 0.02 for the singlet state of species **2-all-IV** with no ligand at CASSCF level of theory....25

Figure S18. Active space natural orbitals (isovalue=0.04) with occupation numbers (in parenthesis) greater than 0.02 for the septet state of species **2-*cis*** with no ligand at CASSCF level of theory. 26

Figure S19. Active space natural orbitals (isovalue=0.04) with occupation numbers (in parenthesis) greater than 0.02 for the quintet state of species **2-*cis*** with no ligand at CASSCF level of theory. 27

Figure S20. Active space natural orbitals (isovalue=0.04) with occupation numbers (in parenthesis) greater than 0.02 for the triplet state of species **2-*cis*** with no ligand at CASSCF level of theory. 28

Figure S21. Active space natural orbitals (isovalue=0.04) with occupation numbers (in parenthesis) greater than 0.02 for the singlet state of species **2-*cis*** with no ligand at CASSCF level of theory. 29

S.3 Supplementary Table....30

Table S1. NTO for the IVCT of compound **2-*cis***. Color scheme: V(III) in blue, V(IV) in purple, V(V) in orange, V(II) in black and delocalization Vanadium center in red. 30

Table S2. NTO for the IVCT of compound **2-*trans***. Color scheme: V(III) in blue, V(IV) in purple, V(V) in orange, V(II) in black, and delocalized vanadium centers in red. 32

Table S3. NTO for the IVCT of compound **2-MeO-*All-IV***. Color scheme: V(III) in blue, V(IV) in purple, V(V) in orange, and delocalized vanadium centers in red. 34

Table S4. Distances in Angstroms between the central μ_6-oxo and the vanadium centers of species with no ligand. 36

Table S5. Energies in kcal/mol relative to the ground state for the **2-*trans*** geometry with different ligands at CASSCF and CASPT2 level of theory. 37

Table S6. Energies in kcal/mol relative to the ground state for the **2-*all-IV*** geometry with different ligands at CASPT2 level of theory..38
Table S7. Energies in kcal/mol relative to the ground state for the **2-*cis*** geometry with different ligands at CASPT2 level of theory. 39
Table S8. Energies in kcal/mol relative to the lowest state for compound **2** at CASSCF level of theory. 40
Table S9. Energies in kcal/mol relative to the lowest state compound **2** ligand at CASPT2 level of theory...41
Table S10. LoProp charges and Mulliken spin density of vanadium centers the septet states for **2-*cis***, **2-*all-IV***, and **2-*trans*** compounds..42
Table S11. LoProp charges and Mulliken spin density of vanadium centers the quintet states for species **2-*cis***, **2-*all-IV***, and **2-*trans*** compounds. 43
Table S12. LoProp charges and Mulliken spin density of vanadium centers the triplet states for 2-cis, 2-all-IV, and 2-trans compounds...44
Table S13. LoProp charges of vanadium centers the singlet states for species **2-*cis***, **2-*all-IV***, and **2-*trans***. 45
Table S14. Hirshfeld Fragmentation Analysis of Hole P_h and Electron P_e for 5 →1 CTDR excitation at λ = 667.5 nm in the **2-MeO-*all-IV*** topology................................46
Table S15. Hirshfeld Fragmentation Analysis of Hole P_h and Electron P_e for 4 →1 CTDR excitation at λ = 642.5 nm in the **2-*trans*** topology...47
Table S16. Hirshfeld Fragmentation Analysis of Hole P_h and Electron P_e for 2 →1 CTDR excitation at λ = 562.0 nm in the **2-MeO-*all-IV*** topology.................................48
Table S17. Hirshfeld Fragmentation Analysis of Hole P_h and Electron P_e for 2-sites 1 →1 IVCT excitation at λ = 504.0 nm in the **2-*trans*** topology....................................49
Table S18. Hirshfeld Fragmentation Analysis of Hole P_h and Electron P_e for 4-sites DDR excitation at λ = 531.1 nm in the **2-MeO-*all-IV*** topology..................................50
Table S19. Hirshfeld Fragmentation Analysis of Hole P_h and Electron P_e for 3-sites DDR excitation at λ = 823.0 nm in the **2-*trans*** topology..51
Table S20. Hirshfeld Fragmentation Analysis of Hole P_h and Electron P_e for 2-sites DDR excitation at λ = 516.2 nm in the **2-MeO-*All-IV*** topology. 52
References 53

S.1 Computational Details

S1.1 Density Functional Theory Calculations

Density Functional Theory (DFT) calculations were performed using the ORCA 6.0.1. package[1, 2] with the PBE0 exchange–correlation functional[3, 4] in conjunction with BS1 basis set (def2-TZVP basis set for the vanadium centers and def2-SVP for all other atoms).[5] All species with unpaired electrons were considered to be in their high spin state indicating the highest possible multiplicity between the vanadium *3d*. Dispersion effects were included using Grimme's D4 correction[6]. The nature of all stationary points and transition states were verified by the calculation of analytical vibrational frequencies. Solvent effects were included using the SMD solvation model[7] with tetrahydro furan (THF) parameters during the optimization process. All calculations were performed without symmetrical constraints.

S1.2 Verification of the Computational Approach

Previous benchmarking studies have shown that hybrid functionals, particularly PBE0, provide reliable descriptions of both ground-state electronic structures and excited-state properties in polyoxometalates.[8, 9] To further validate the functional, we reproduced the electronic absorption spectrum of the well-characterized cluster **$[(V_6O_5)(\mu_6\text{-}O)(\mu_2\text{-}OCH_3)_{12}]^{-}$**. Formation of an oxygen vacancy generates the established V(III) defect site while the remaining centers become V(IV); the resulting electronic absorption spectrum exhibits a characteristic band near 526 nm and a shoulder at higher energy around 400 nm.[10] The TDDFT calculations with def2-TZVP for all atoms utilized the geometry obtained with BS1 basis set level theory, Figure S1 illustrate reproduce these features, giving a peak at λ = 520.3 nm assigned to $d_{xy}\rightarrow d_{x^2-y^2}$ transition and λ = 401.5 nm for the 2V(IV) → V(III) IVCT. These results support the accuracy of the chosen computational protocol, and all IVCT analyses in this work were performed at the SMD(THF)-PBE0/def2-TZVP-D4//BS1 level of theory.

S1.3 Complete Active Space Calculations

Multiconfigurational complete active space self-consistent field (CASSCF) calculations followed by second-order perturbation theory (CASPT2) were performed on the $[(V_6O_5)(\mu_6\text{-}O)(\mu_2\text{-}OCH_3)_{12}]$ DFT optimized geometries using the OpenMolcas software suite.[11-13] An active space of 6 electrons in 18 orbitals (6e, 18o) was used. This includes the three lowest 3d orbitals for each of the vanadium centers and all the corresponding vanadium 3d electrons. This choice is based on the quasi-octahedral ligand environment of the metal centers that will lead to a large crystal field splitting. For CASPT2 calculations, an imaginary shift of 0.2 au and the so-called IPEA shift of 0.25 were employed.[14, 15] Scalar relativistic effects were included at the CASSCF level using the second order

Douglas–Kroll–Hess Hamiltonian[16, 17] and relativistic all electron ANO-RCC basis sets. An ANO-RCC-VTZP was used in all metal centers, an ANO-RCC-DZVP in all oxygen atoms and atoms in the vanadium first coordination sphere, and ANO-RCC-MB in all other atoms.[18, 19] Specifically, the following contractions were used: [6s,5p,3d,2f,1g] for vanadium, [3s,2p,1d] for oxygen, [2s,1p] for carbon, and [1s] for hydrogen. Cholesky decomposition in conjunction with local-exchange screening was used to reduce the computational cost.[20] For CASPT2 calculations, the default number of frozen orbitals was selected in the correlation space.

S1.4 Influence of Ligand and Charge on the Stability of Redox Topologies

To clarify the influence of ligand identity on the stability of the switching topologies, we expanded the computational study to include several additional ligands spanning a range of σ-donor strengths, *π*-acceptor abilities, and charges. Specifically, three neutral ligands (pyridine, NH_3, and DMAP) and three anionic ligands (CN^-, Cl^-, and OH^-) were examined. The corresponding structures and energetics are presented in Figures S7 and S8.

These additional systems enable a systematic comparison of the relative stability of the delocalized all-V(IV) topology and the localized cis/trans electromers. As summarized in Figure S9, the relative stability is quantified using $\Delta G_{IV\text{-}C/T}$, defined as the Gibbs free energy difference between the *all-IV* state and the lower-energy cis or trans topology. Among the neutral ligands, a qualitative *σ*-donor trend is observed: stronger donors such as DMAP stabilize the *all-IV* topology more effectively than NH_3 or pyridine. A similar trend is seen for oxygen donors, where MeO^- stabilizes the *all-IV* state more strongly than HO^-.

More generally, the calculations indicate that anionic ligands have the strongest influence on stabilizing the delocalized *all-IV* configuration. This behavior can be rationalized by electrostatic effects that destabilize localized configurations involving V(III) (*d²*) centers. For example, while the neutral *π*-acceptor ligand CO strongly destabilizes the *all-IV* topology ($\Delta G_{IV\text{-}C/T}$ = 14.0 kcal mol⁻¹), the anionic *π*-acceptor CN^- yields a much smaller energy difference ($\Delta G_{IV\text{-}C/T}$ = 8–9 kcal mol⁻¹). Similarly, Cl^- produces an intermediate value ($\Delta G_{IV\text{-}C/T}$ ≈ 3.1 kcal mol⁻¹), bringing the delocalized and localized states closer in energy but still favoring the localized topology.

Full electronic-state switching occurs only for strongly donating anionic ligands, such as MeO^- and HO^-, which stabilize the *all-IV* state as the thermodynamic ground state with $\Delta G_{IV\text{–}C/T}$ values of −11.2 and −7.8 kcal mol⁻¹, respectively. These results indicate that both ligand charge and *σ*-donor strength contribute to tuning the balance between localized and delocalized electronic structures.

In terms of the relative stability of the localized electromers, the *trans* topology is generally lower in energy than the *cis* topology across the ligand set examined. The only exception

is observed for the CN^- ligand, where the *cis* configuration is slightly more stable. This difference likely reflects the distinct electronic characteristics of CN^-, which combines σ-donor, π-acceptor, and anionic features.

S1.5 Assignment of intervalence charge transfer

Assignment of intervalence charge transfer (IVCT) transitions as either charge-transfer-driven redistribution (CTDR) or delocalization-driven redistribution (DDR) can initially be inferred from visualization of the Natural Transition Orbitals (NTOs),[21] as shown in Tables S1–S3. In the CTDR mechanism, the hole orbital (HOTO) and electron orbital (LUTO) display a clear donor–acceptor pattern, with electron density removed from one set of metal centers and accumulated at a specific acceptor site. In contrast, DDR transitions exhibit more distributed electron density across multiple vanadium centers, reflecting a delocalized multi-center excitation.

To provide a quantitative descriptor for this assignment, we further analyze the excitations using Hirshfeld fragmentation analysis[22, 23] derived from the TDDFT results and implemented through the Multiwfn package.[24, 25] This analysis quantifies the contribution of each atomic fragment to the excitation and therefore enables a reproducible classification of CTDR and DDR transitions.

In this framework, the normalized quantities P_h% and P_e% represent the origin and destination of electron density during the excitation. Specifically, P_h% describes the fragment from which electron density is removed (hole contribution), while P_e% indicates the fragment where electron density accumulates (electron contribution). These values therefore identify the specific metal centers involved in the redistribution process.

As illustrated in Tables S14 and S18, CTDR transitions are characterized by strong localization of electron density at the acceptor center, typically with P_e% values around 65–70%, while the donor centers are largely depleted after the transition. In contrast, there is no clear donor and acceptor site in DDR, and the transitions display a more distributed electron density among several vanadium centers, with no single center exhibiting a dominant P_e% contribution greater than 50%.

To examine the generality of this metric, representative CTDR transitions involving different numbers of active metal centers (5→1, 4→1, 2→1, and 1→1) are presented in Tables S14–S17. These examples show that the classification remains consistent regardless of the number of participating metal sites. Similarly, Tables S18–S19 illustrate DDR transitions in which electron density redistribution is distributed across four-, three-, and two-center manifolds.

The change in electron density, defined as ΔP (%) = $P_h - P_e$, provides a practical metric to distinguish CTDR from DDR. In CTDR, the acceptor site, typically identified as the center with the largest ΔP in the table (Tables S14–S17), consistently shows ΔP values

of no less than 40%. In contrast, DDR does not exhibit a well-defined acceptor site, and no center shows ΔP values exceeding 35%. These values can serve as a useful working threshold for differentiating the two mechanisms. However, the criteria derived here for pristine vanadium systems may not be directly generalizable to other systems and should be applied with appropriate caution.

It should be noted that, in most cases, visualization of the HOTO and LUTO provides assignments consistent with the fragmentation analysis when these orbitals account for a large fraction of the transition. However, in cases where the HOTO→LUTO contribution is not dominant, additional frontier orbitals (e.g., HOTO−1 or LUTO+1) should also be considered to fully capture the excitation character.

S.2 Supplementary Figures

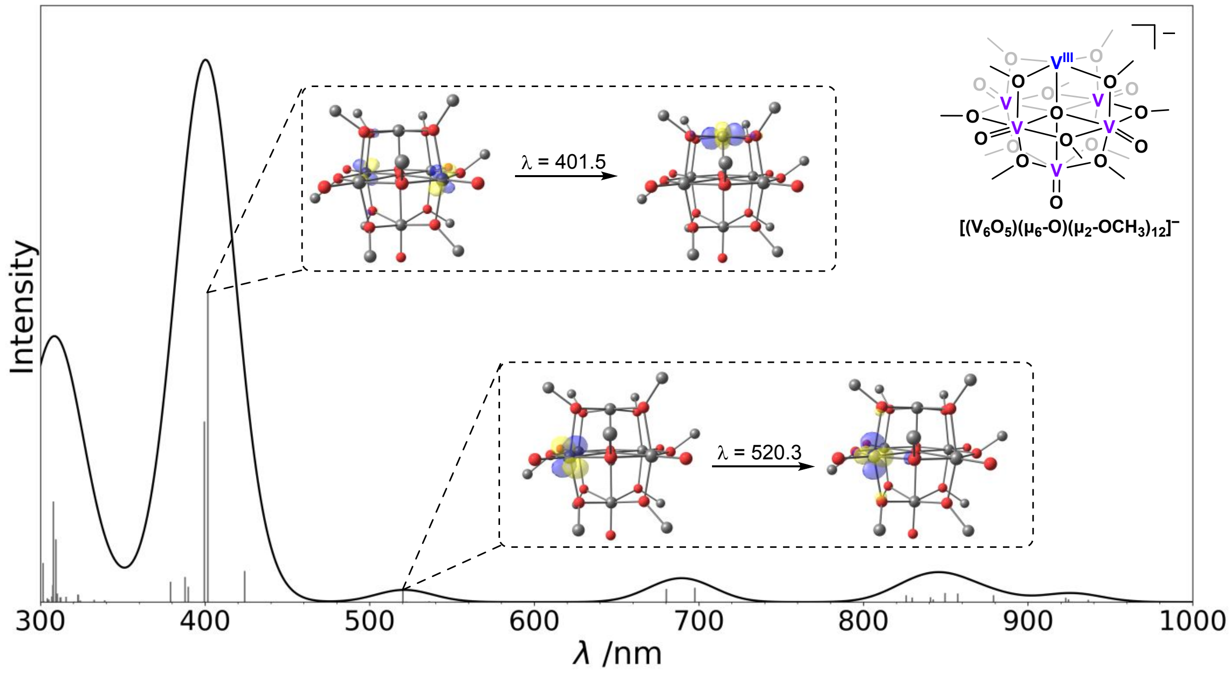

Figure S1. Simulated electronic absorption spectrum of **$[(V_6O_5)(\mu_6\text{-}O)(\mu_2\text{-}OCH_3)_{12}]^-$** at SMD(THF)-PBE0/def2-TZVP-D4//BS1 level theory, with selected NTOs for key transitions.

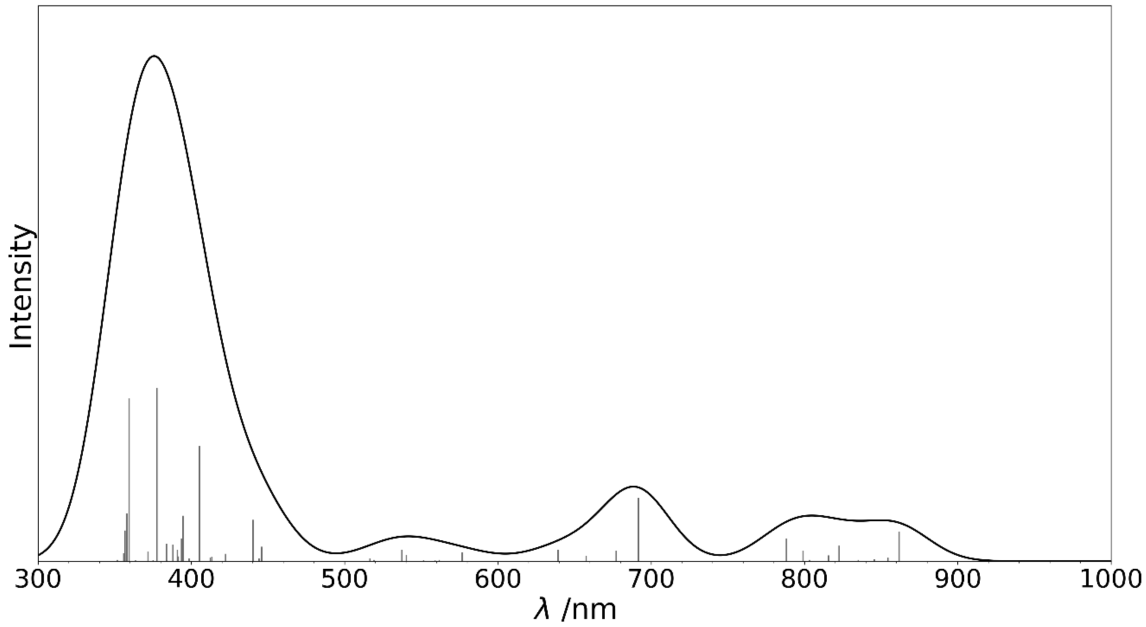

Figure S2. Simulated electronic absorption spectrum of compound ***2-cis**.*

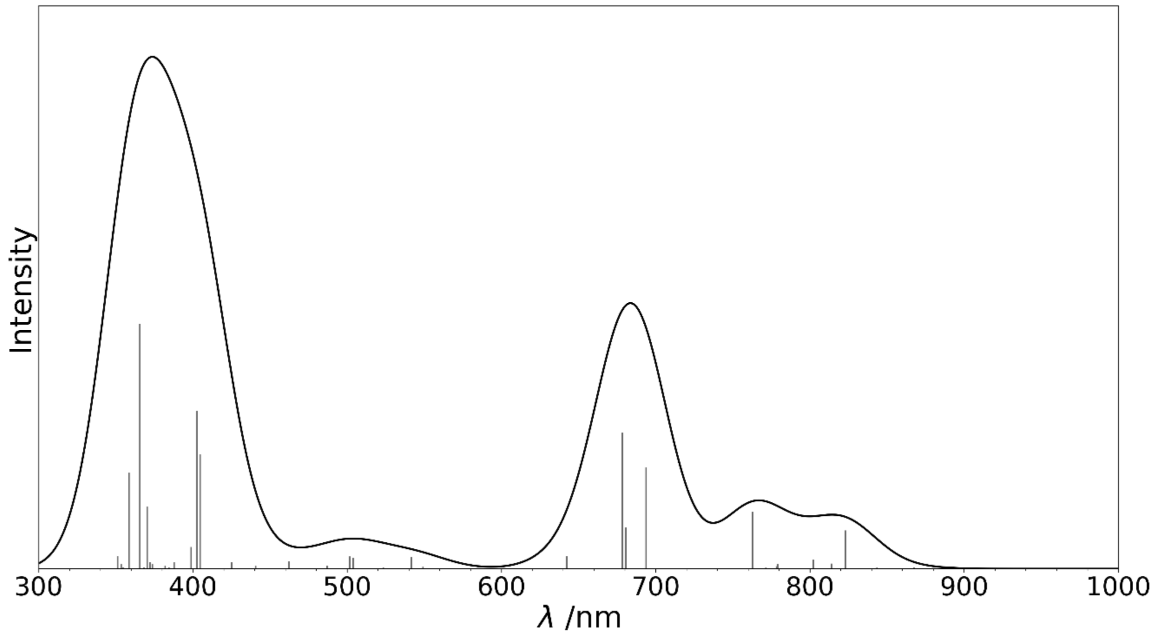

Figure S3. Simulated electronic absorption spectrum of compound **2-*trans***.

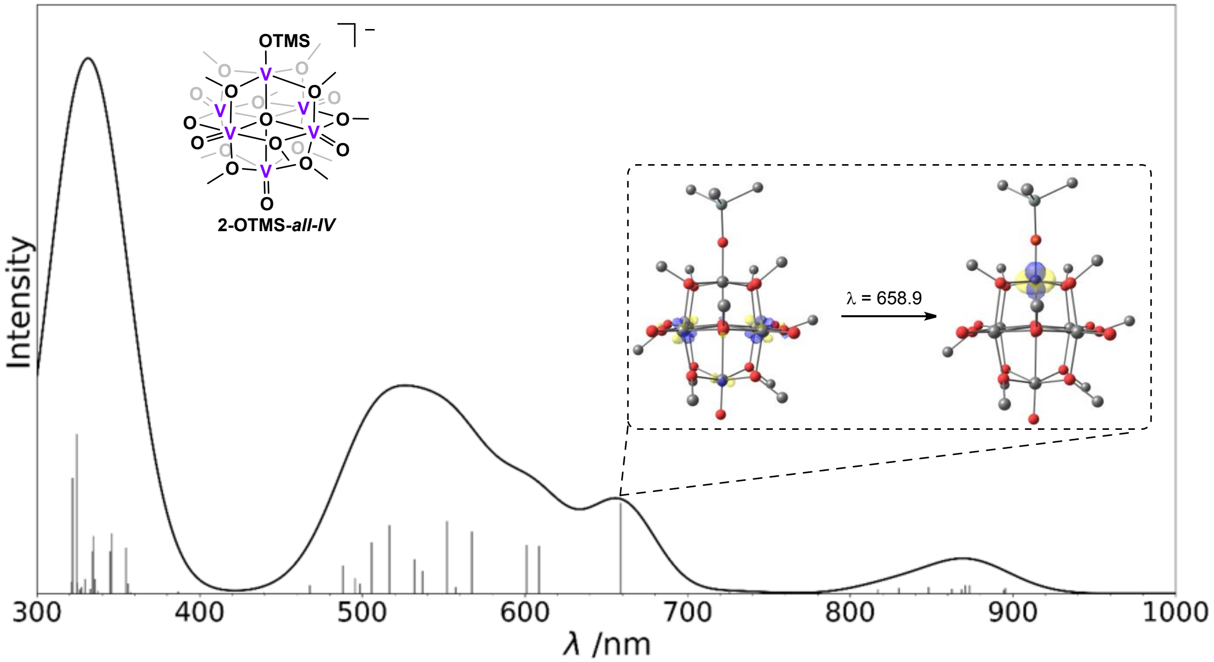

Figure S4. Simulated electronic absorption spectrum of compound **2-OTMS-*All-IV*.**

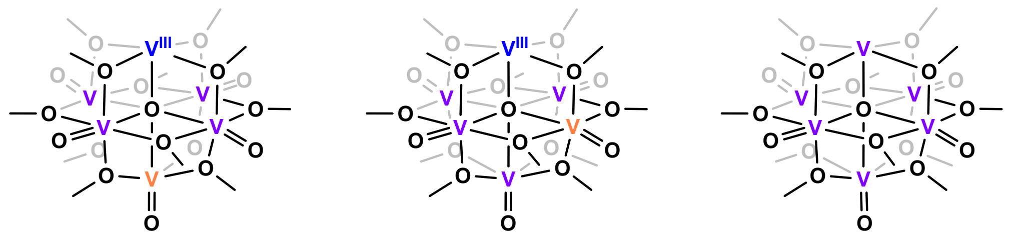

2-trans **0.0** | ***2-cis*** **0.5** | ***2-all-IV*** **7.5**

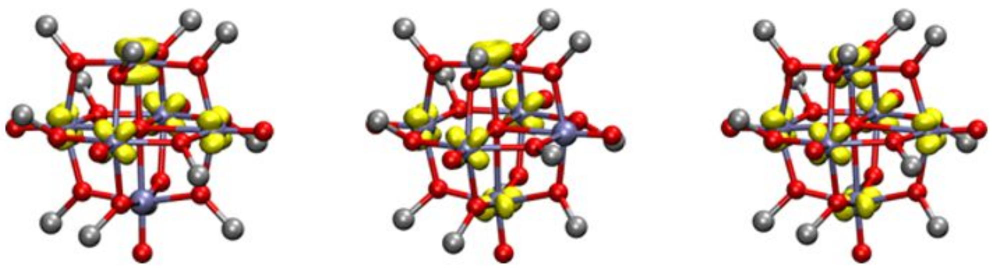

Figure S5. Schematic representation of different redox topologies of the oxygen-deficient **2** species (top) and DFT spin density (bottom). Color scheme: V(**III**) in blue, V(**IV**) in purple, V(V) in orange, and spin density in yellow. Relative Gibbs free energies are in kcal/mol. Hydrogen atoms were omitted for clarity.

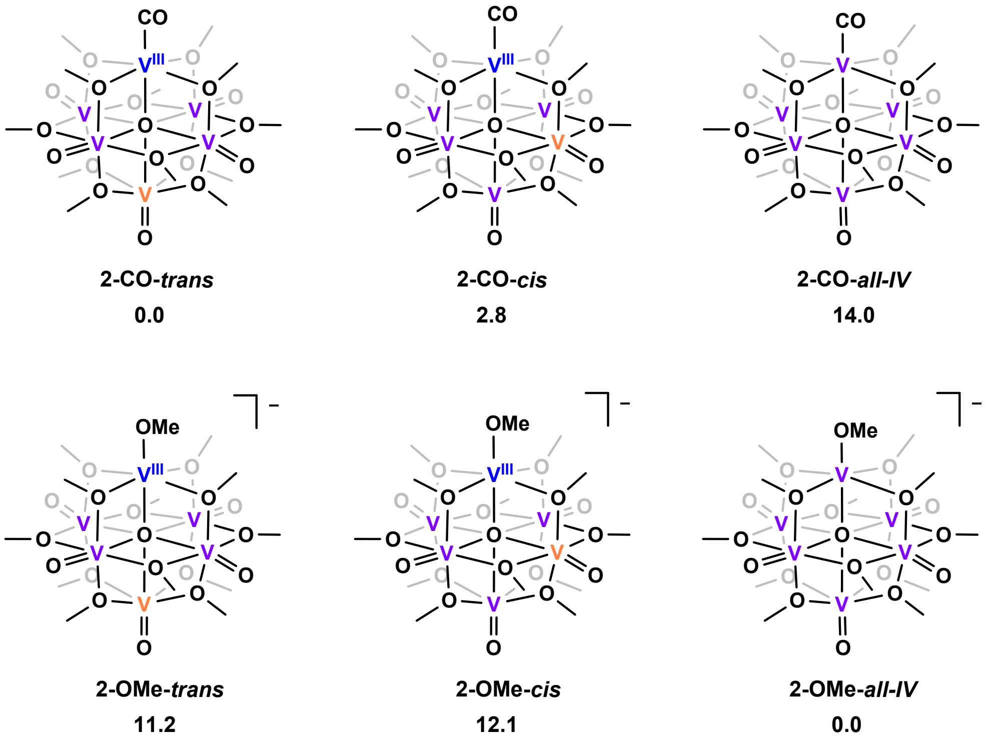

Figure S6. Different redox topologies of **2-CO** (top) and **2-OMe** (bottom) species. Relative Gibbs free energies are in kcal/mol. Color scheme: V(III) in red, V(IV) in blue, and V(V) in orange.

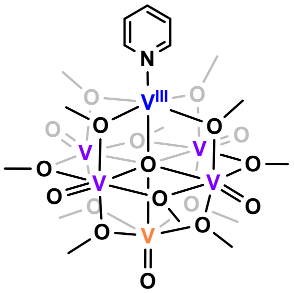

2-Py-*trans*
0.0

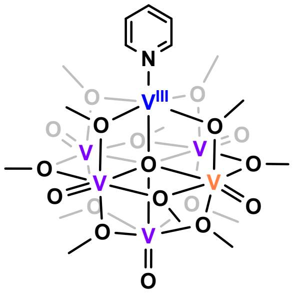

2-Py-*cis*
2.3

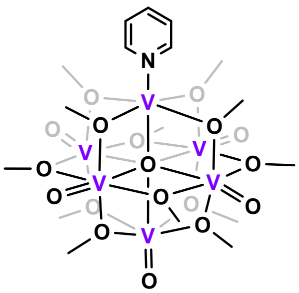

2-Py-*all-IV*
10.1

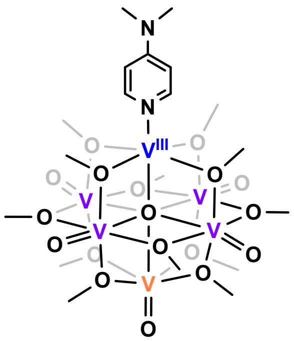

2-DMAP-*trans*
0.0

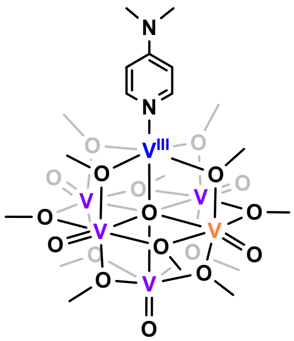

2-DMAP-*cis*
0.3

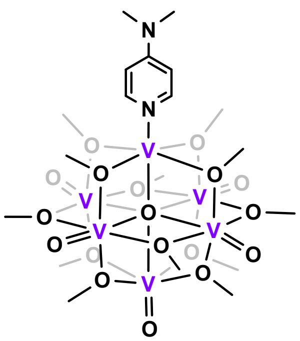

2-DMAP-*all-IV*
9.0

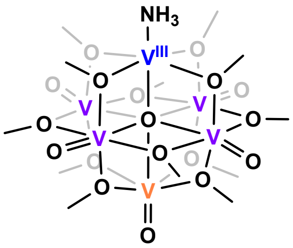

2-NH_3-*trans*
0.0

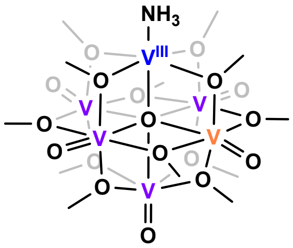

2-NH_3-*cis*
2.5

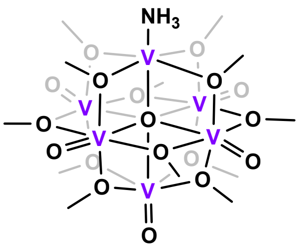

2-NH_3-*all-IV*
10.2

Figure S7. Relative stabilities of trans, *cis*, and *all-IV* topologies influenced by selected neutral ligands. Relative Gibbs free energies are given in kcal/mol. Color scheme: V(III) in red, V(IV) in blue, and V(V) in orange.

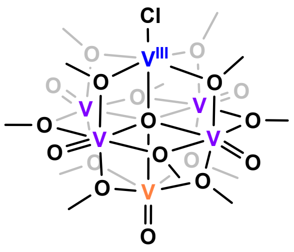

2-Cl-*trans*
0.0

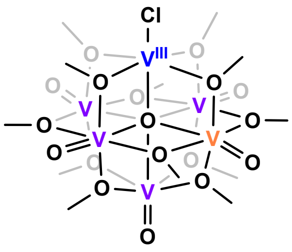

2-Cl-*cis*
0.5

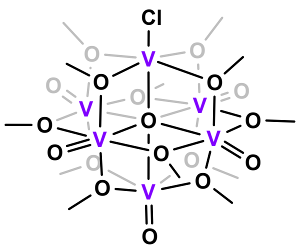

2-Cl-*all-IV*
3.1

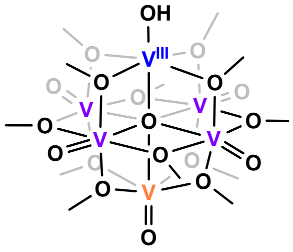

2-OH-*trans*
7.8

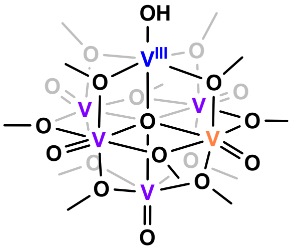

2-OH-*cis*
8.7

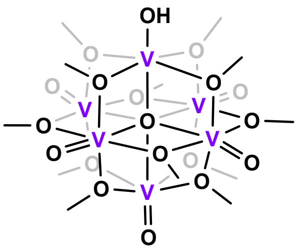

2-OH-*all-IV*
0.0

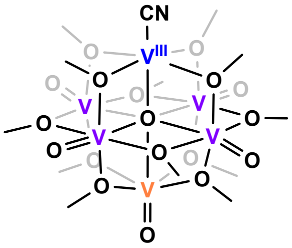

2-CN-*trans*
1.0

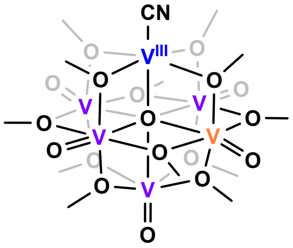

2-CN-*cis*
0.0

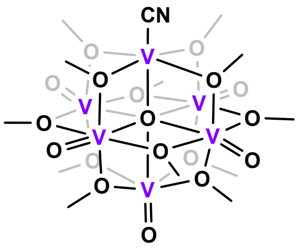

2-CN-*all-IV*
8.2

Figure S8. Relative stabilities of *cis*, *trans*, and *all-IV* topologies influenced by selected anionic ligands. Relative Gibbs free energies are given in kcal/mol. Color scheme: V(III) in red, V(IV) in blue, and V(V) in orange.

	Neutral ligand				*Anionic ligand*			
**Ligand**	CO	NH_3	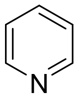	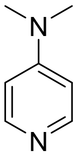	^{-}CN	^{-}Cl	^{-}OH	^{-}OMe
**$\Delta G_{IV\text{-}C/T}$**	14.0	10.2	9.3	9.0	8.2	3.1	-7.8	-11.2
**$\Delta G_{C\text{-}T}$**	2.8	2.5	2.3	0.3	-1.0	0.5	0.8	0.8

Figure S9. Gibbs free energy (kcal/mol) comparison of redox topologies influenced by various ligands. ΔGIV-C/T is defined as the energy difference between the *all-IV* state and the lower-energy *cis* or *trans* topology, and ΔGC-T is the energy difference between the cis and trans topologies.

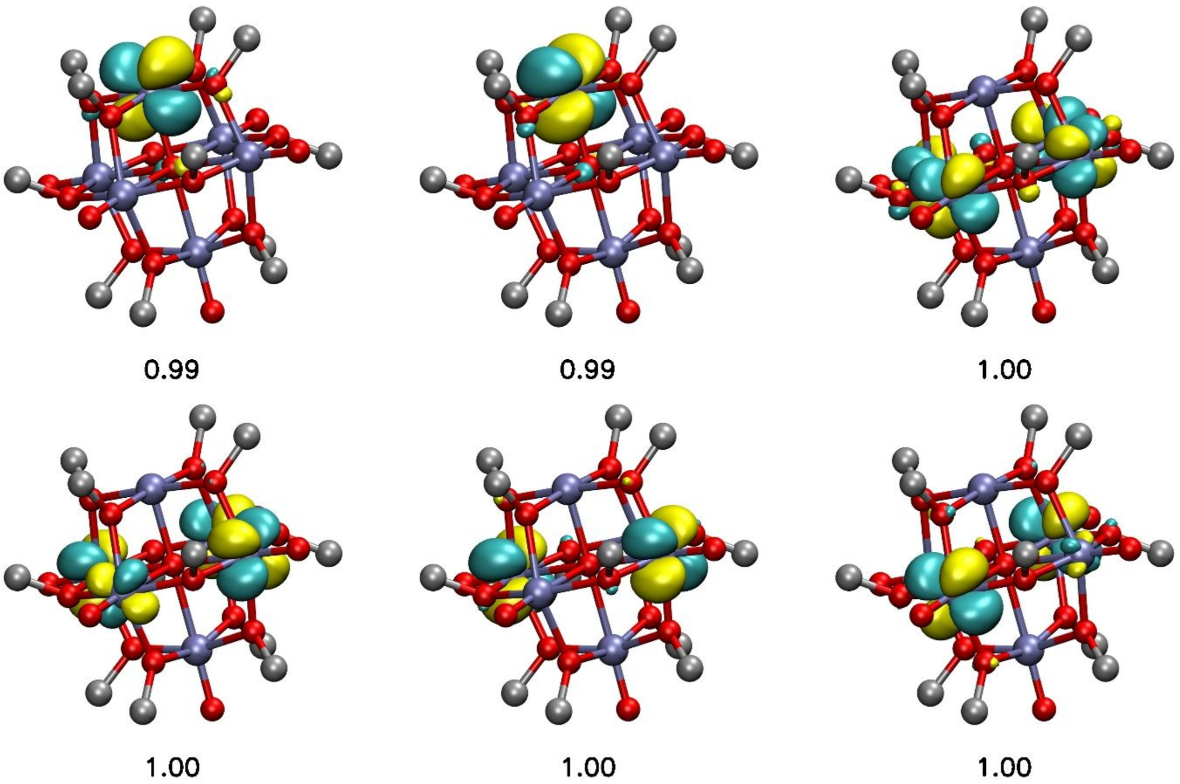

Figure S10. Active space natural orbitals (isovalue=0.04) with occupation numbers (in parenthesis) greater than 0.02 for the septet state of species **2-*trans*** with no ligand at CASSCF level of theory. Orbital occupations are shown below the orbitals. Color code: Vanadium in light purple, oxygen in red, and carbon in grey. Hydrogens are not shown for clarity.

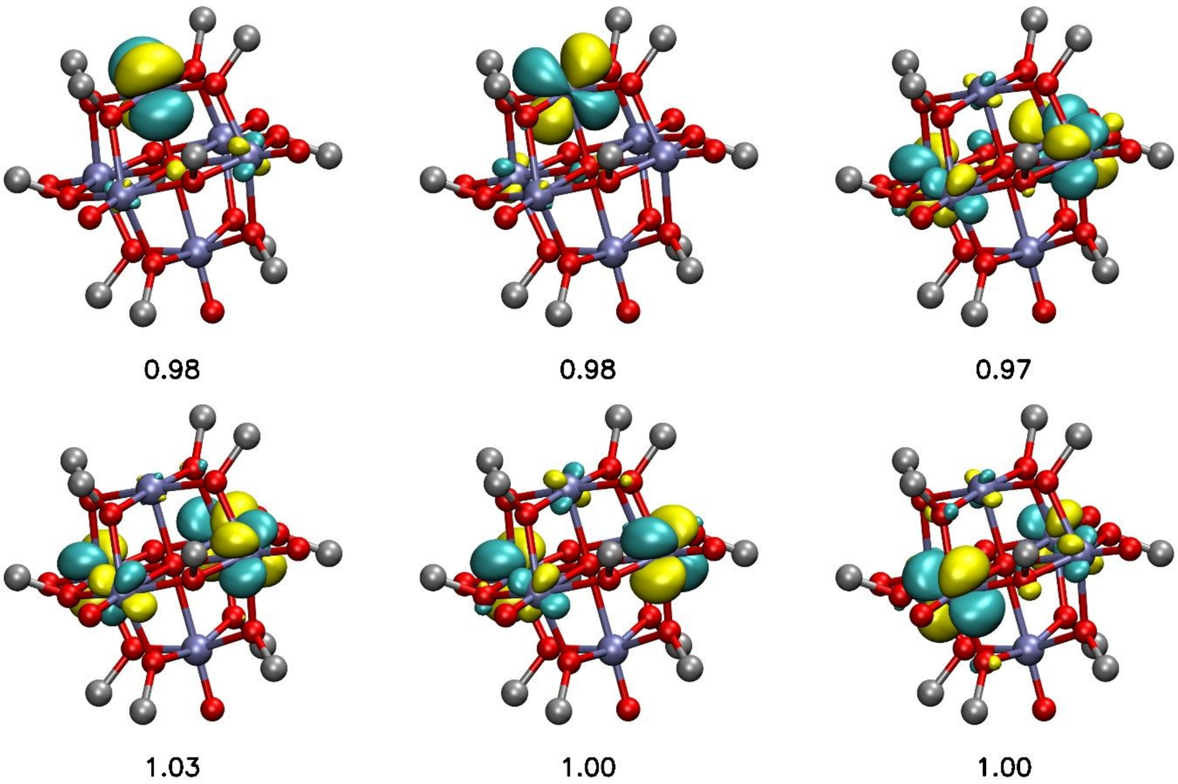

Figure S11. Active space natural orbitals (isovalue=0.04) with occupation numbers (in parenthesis) greater than 0.02 for the quintet state of species **2-*trans*** with no ligand at CASSCF level of theory. Orbital occupations are shown below the orbitals. Color code: Vanadium in light purple, oxygen in red, and carbon in grey. Hydrogens are not shown for clarity.

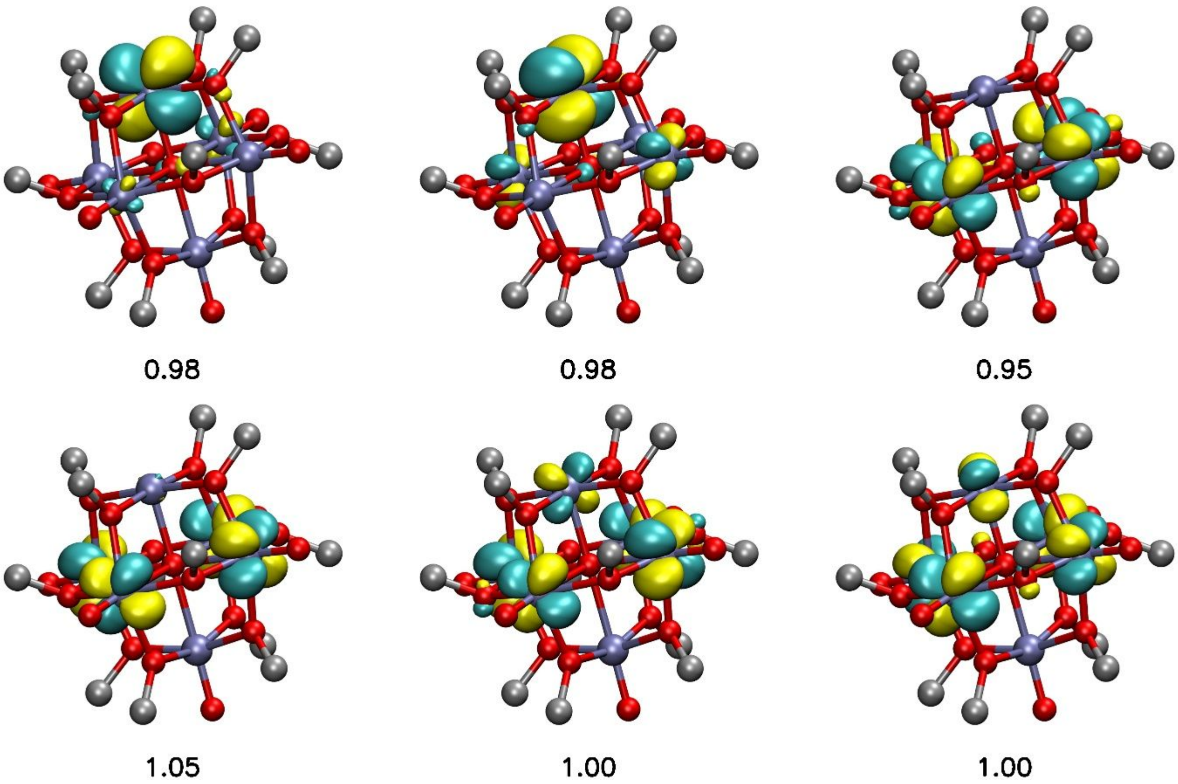

Figure S12. Active space natural orbitals (isovalue=0.04) with occupation numbers (in parenthesis) greater than 0.02 for the triplet state of species **2-*trans*** with no ligand at CASSCF level of theory. Orbital occupations are shown below the orbitals. Color code: Vanadium in light purple, oxygen in red, and carbon in grey. Hydrogens are not shown for clarity.

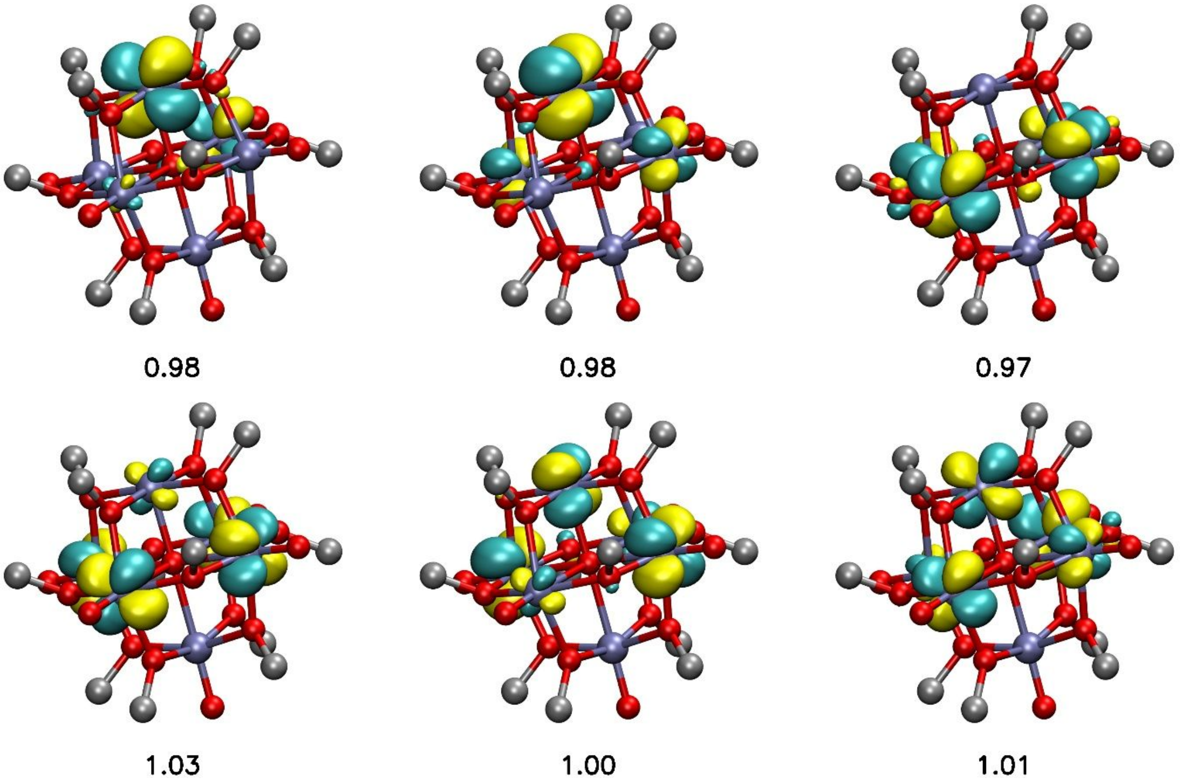

Figure S13. Active space natural orbitals (isovalue=0.04) with occupation numbers (in parenthesis) greater than 0.02 for the singlet state of species **2-*trans*** with no ligand at CASSCF level of theory. Orbital occupations are shown below the orbitals. Color code: Vanadium in light purple, oxygen in red, and carbon in grey. Hydrogens are not shown for clarity.

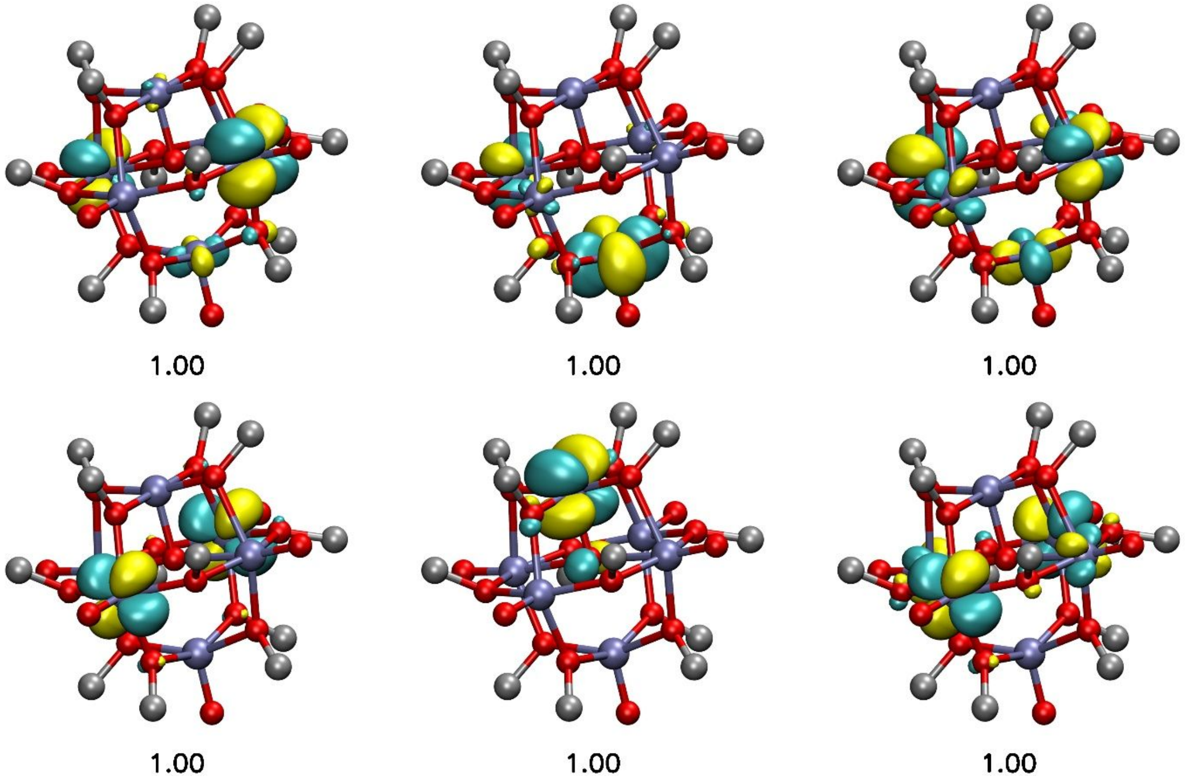

Figure S14. Active space natural orbitals (isovalue=0.04) with occupation numbers (in parenthesis) greater than 0.02 for the septet state of species **2-*all-IV*** with no ligand at CASSCF level of theory. Orbital occupations are shown below the orbitals. Color code: Vanadium in light purple, oxygen in red, and carbon in grey. Hydrogens are not shown for clarity.

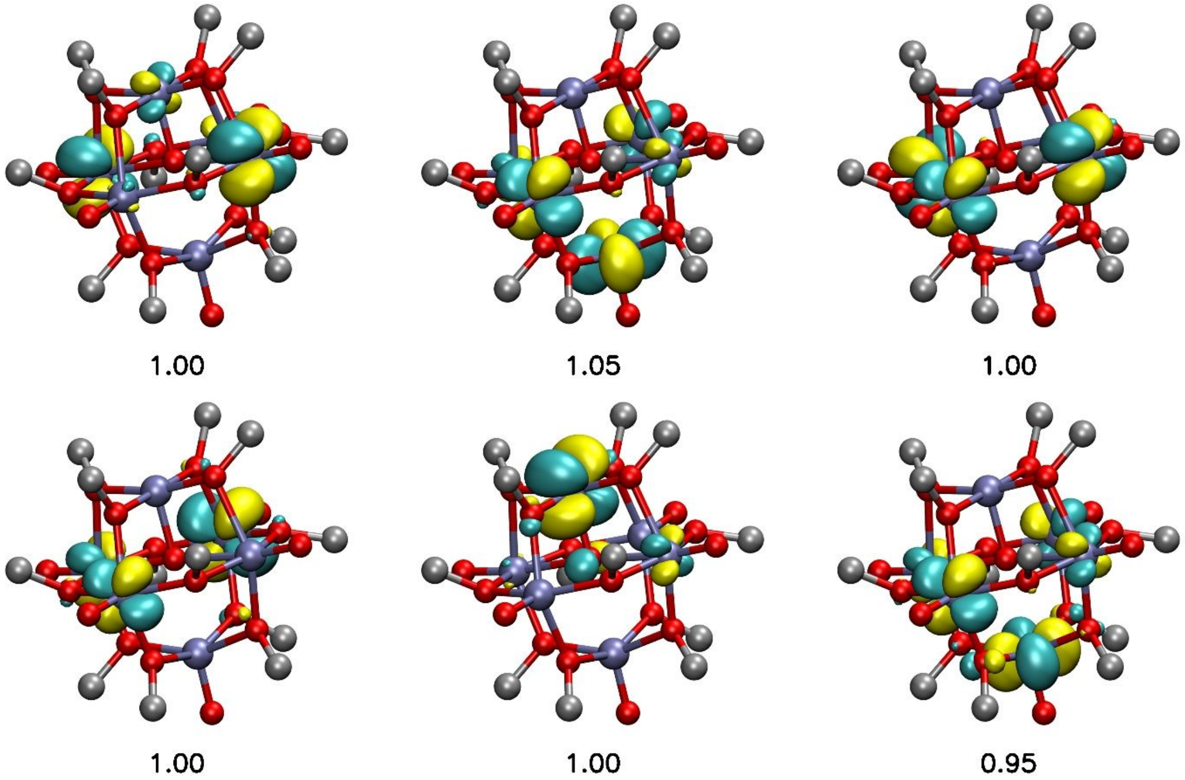

Figure S15. Active space natural orbitals (isovalue=0.04) with occupation numbers (in parenthesis) greater than 0.02 for the quintet state of species ***2-all-IV*** with no ligand at CASSCF level of theory. Orbital occupations are shown below the orbitals. Color code: Vanadium in light purple, oxygen in red, and carbon in grey. Hydrogens are not shown for clarity.

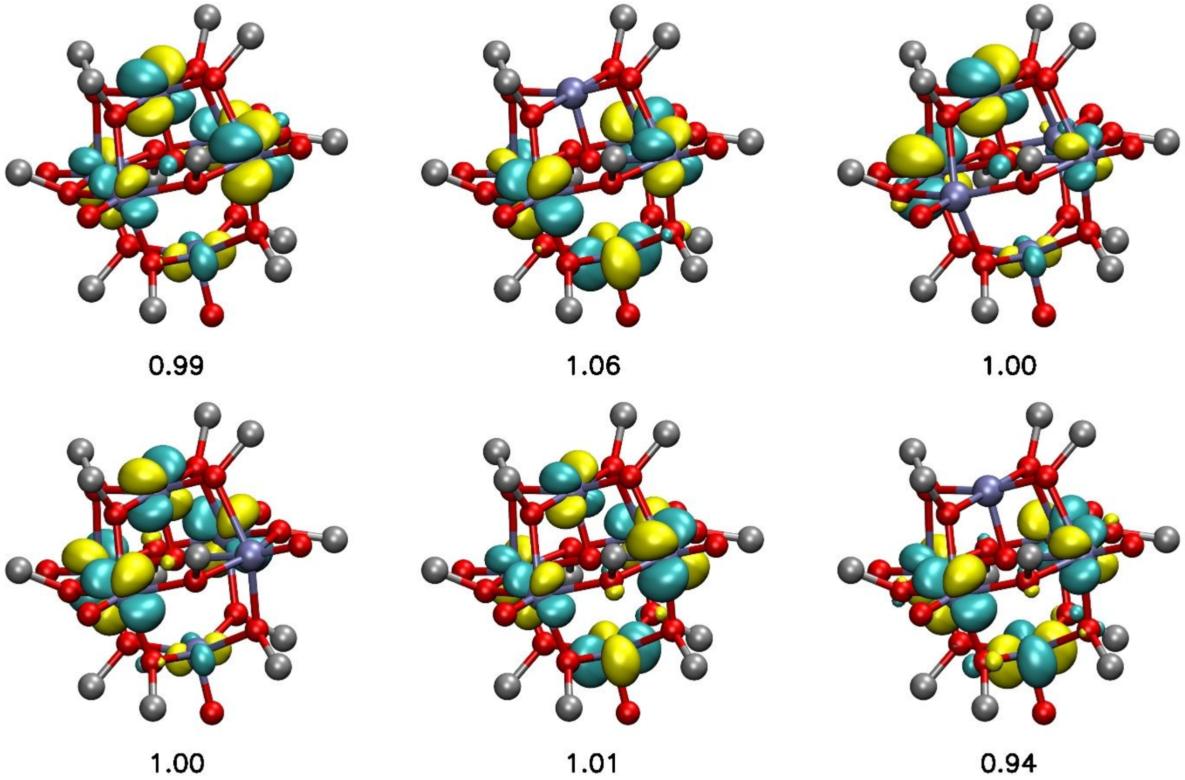

Figure S16. Active space natural orbitals (isovalue=0.04) with occupation numbers (in parenthesis) greater than 0.02 for the triplet state of species ***2-all-IV*** with no ligand at CASSCF level of theory. Orbital occupations are shown below the orbitals. Color code: Vanadium in light purple, oxygen in red, and carbon in grey. Hydrogens are not shown for clarity.

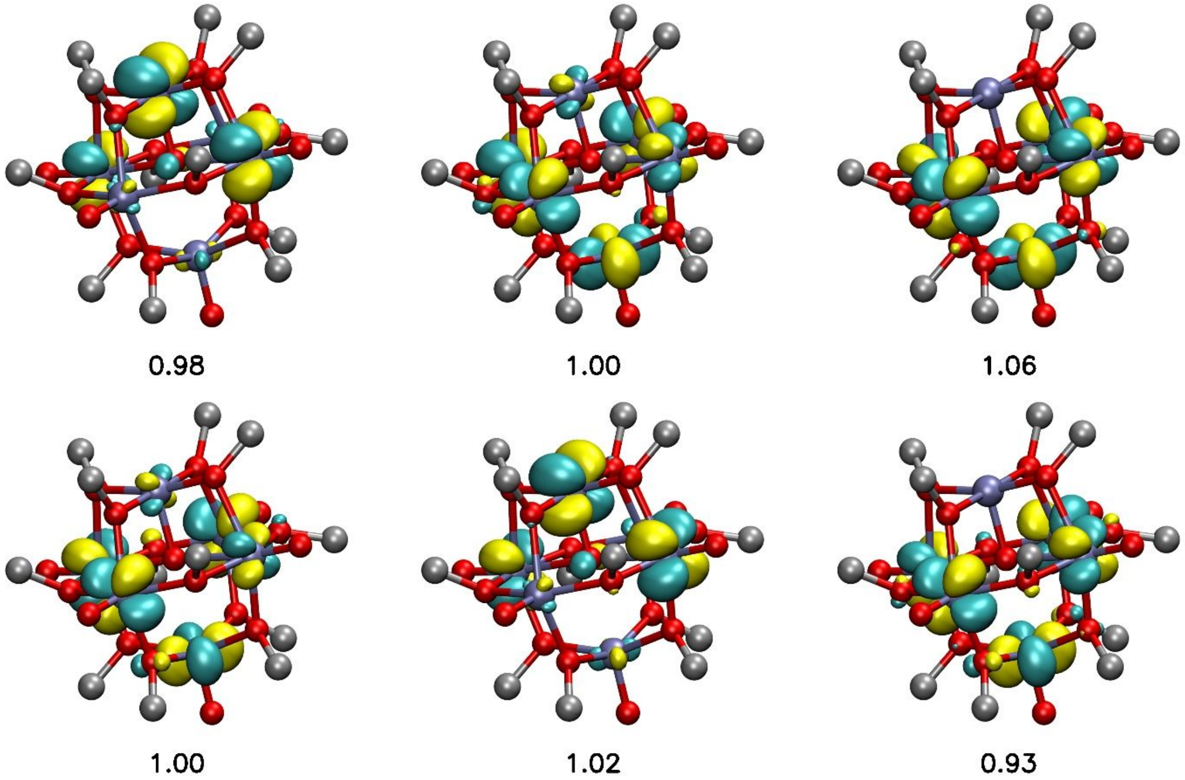

Figure S17. Active space natural orbitals (isovalue=0.04) with occupation numbers (in parenthesis) greater than 0.02 for the singlet state of species **2-all-IV** with no ligand at CASSCF level of theory. Orbital occupations are shown below the orbitals. Color code: Vanadium in light purple, oxygen in red, and carbon in grey. Hydrogens are not shown for clarity.

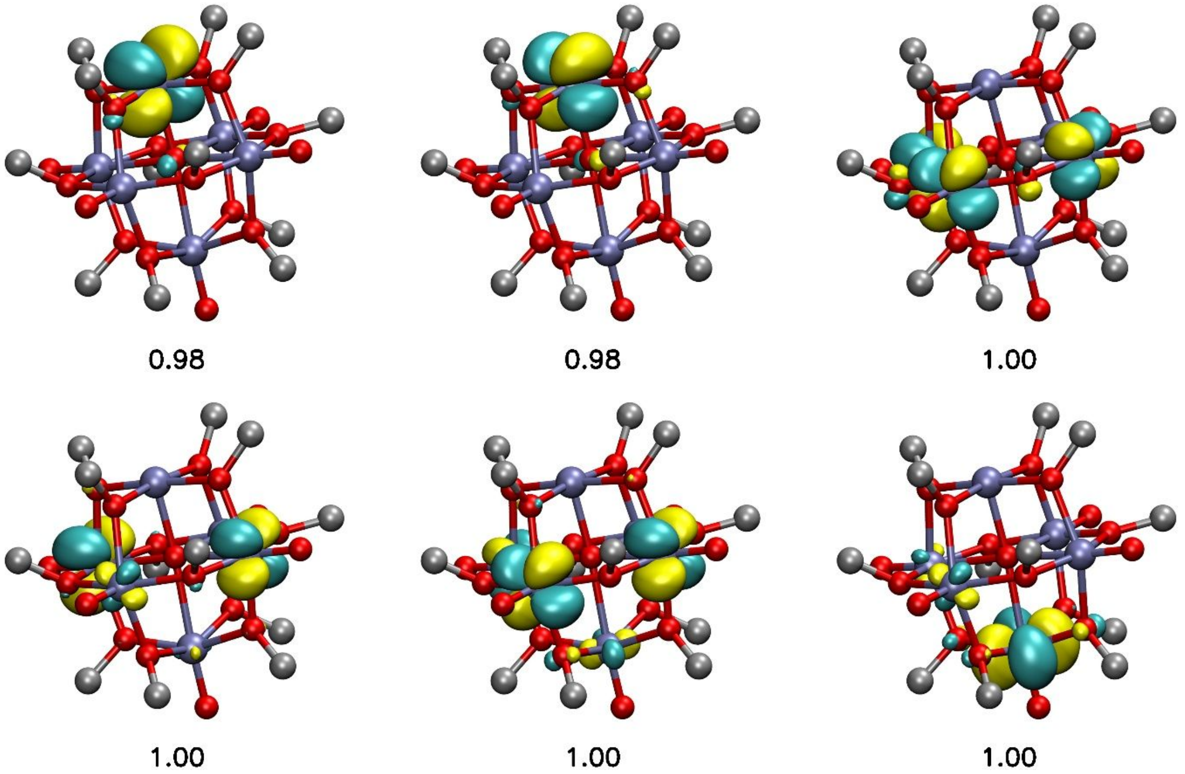

Figure S18. Active space natural orbitals (isovalue=0.04) with occupation numbers (in parenthesis) greater than 0.02 for the septet state of species ***2-cis*** with no ligand at CASSCF level of theory. Orbital occupations are shown below the orbitals. Color code: Vanadium in light purple, oxygen in red, and carbon in grey. Hydrogens are not shown for clarity.

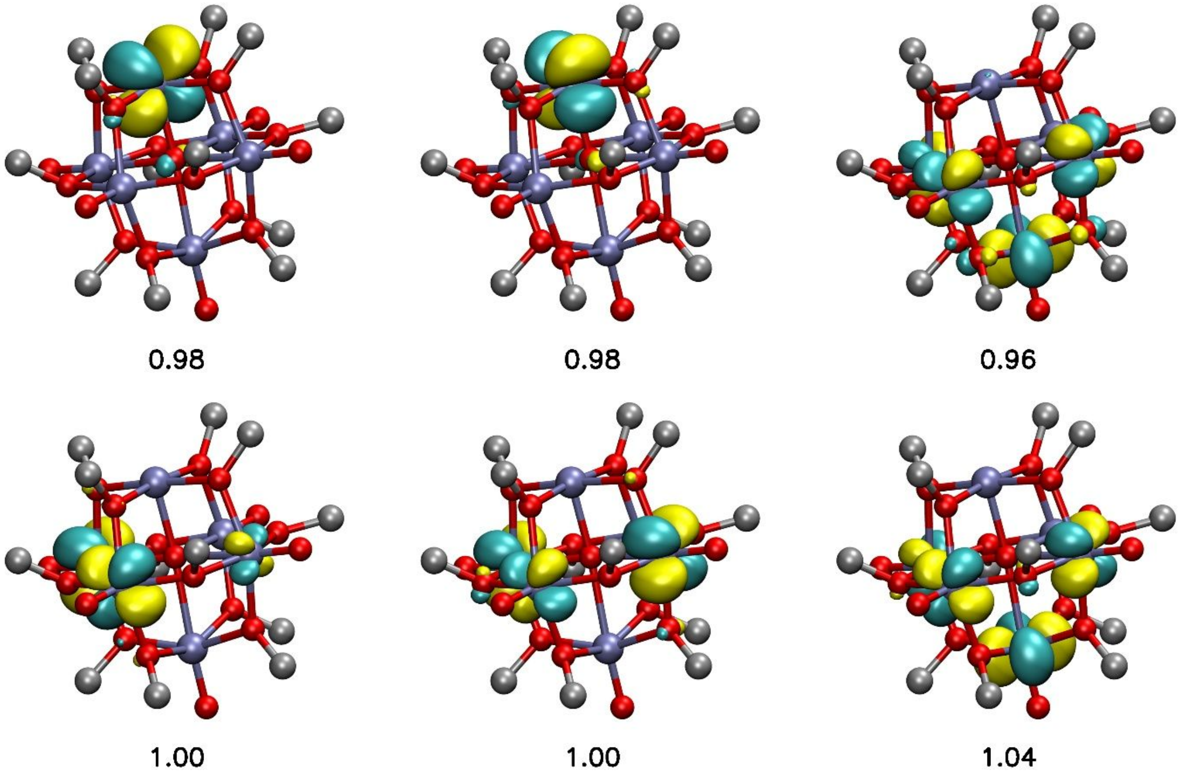

Figure S19. Active space natural orbitals (isovalue=0.04) with occupation numbers (in parenthesis) greater than 0.02 for the quintet state of species **2-*cis*** with no ligand at CASSCF level of theory. Orbital occupations are shown below the orbitals. Color code: Vanadium in light purple, oxygen in red, and carbon in grey. Hydrogens are not shown for clarity.

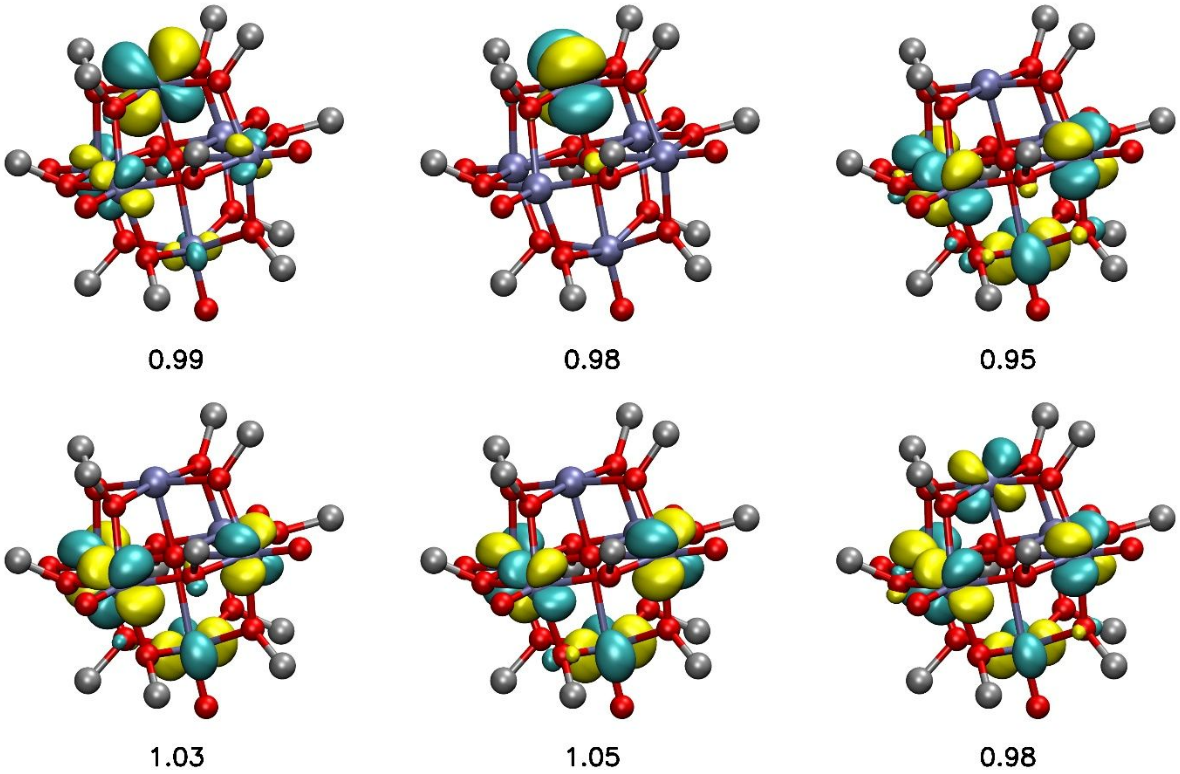

Figure S20. Active space natural orbitals (isovalue=0.04) with occupation numbers (in parenthesis) greater than 0.02 for the triplet state of species ***2-cis*** with no ligand at CASSCF level of theory. Orbital occupations are shown below the orbitals. Color code: Vanadium in light purple, oxygen in red, and carbon in grey. Hydrogens are not shown for clarity.

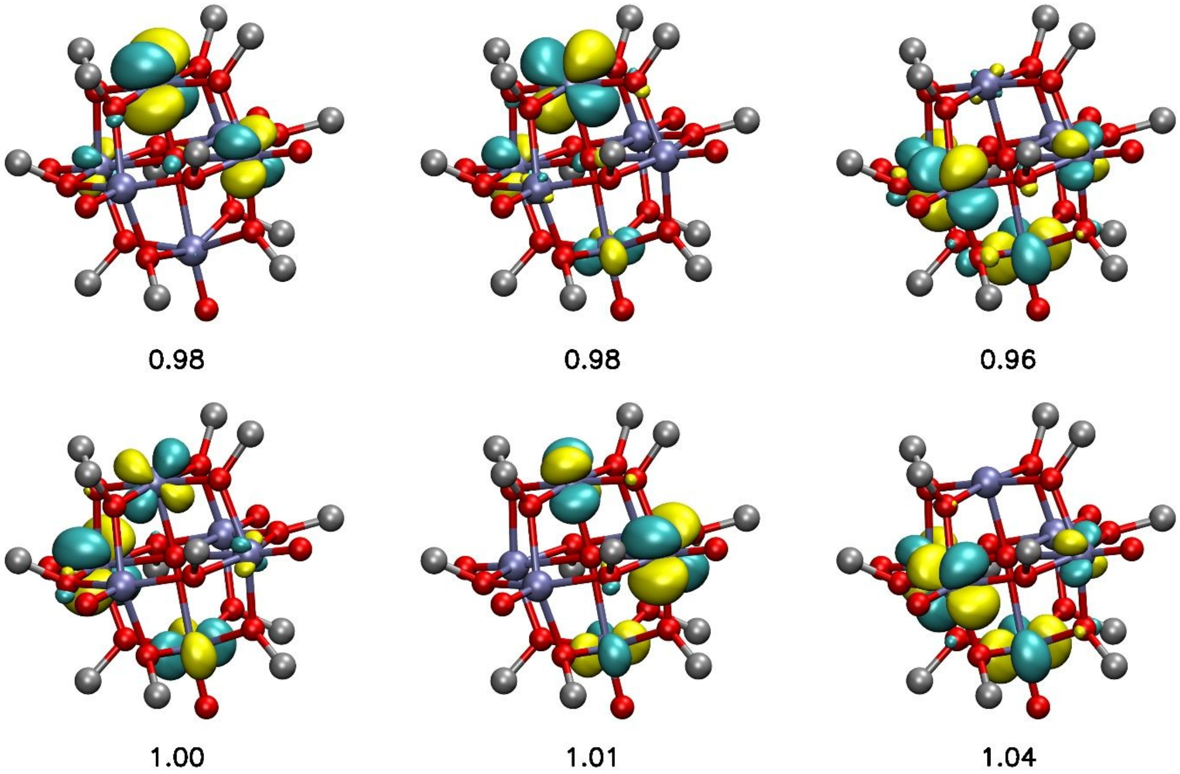

Figure S21. Active space natural orbitals (isovalue=0.04) with occupation numbers (in parenthesis) greater than 0.02 for the singlet state of species ***2-cis*** with no ligand at CASSCF level of theory. Orbital occupations are shown below the orbitals. Color code: Vanadium in light purple, oxygen in red, and carbon in grey. Hydrogens are not shown for clarity.

S.3 Supplementary Table

Table S1. NTO for the IVCT of compound **2-*cis***. Color scheme: V(III) in blue, V(IV) in purple, V(V) in orange, V(II) in black and delocalization Vanadium center in red.

Excitation	HOTO	LUTO	Transition presentation
λ = 861.8 nm	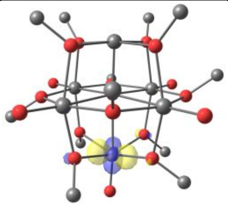	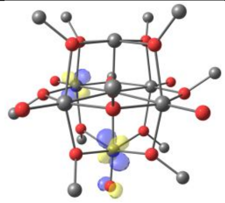	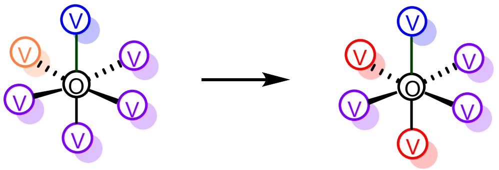 Delocalization-driven redistribution
λ = 799.1 nm	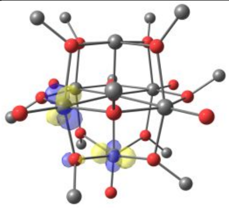	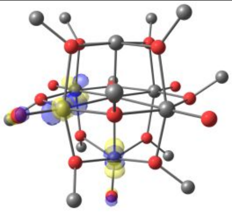	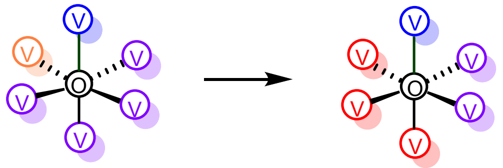 Delocalization-driven redistribution
λ = 691.7 nm	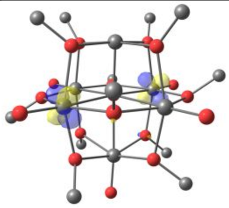	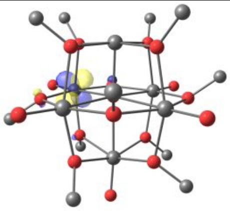	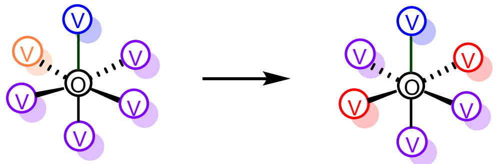 Charge-transfer-driven redistribution
λ = 639.1 nm	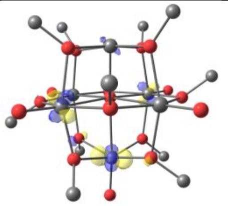	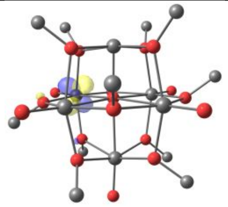	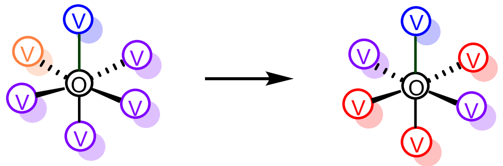 Charge-transfer-driven redistribution
λ = 540.2 nm	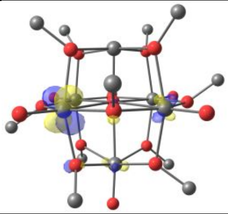	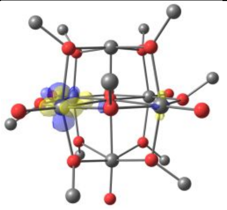	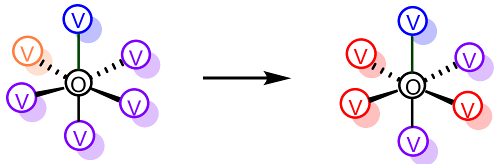 Delocalization-driven redistribution
λ = 537.4 nm	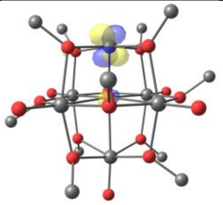	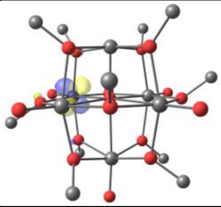	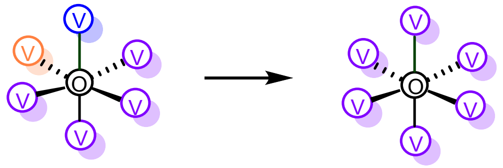 Charge transfers V(III) → V(V)
λ = 445.9 nm	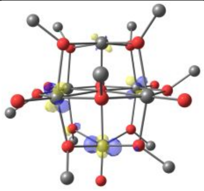	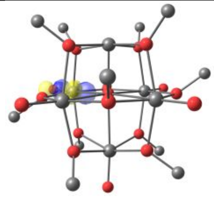	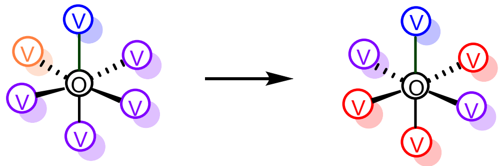 Charge-transfer-driven redistribution

Table S1 (continue). NTO for IVCT of compound **2-*cis***.

λ = 440.4 nm	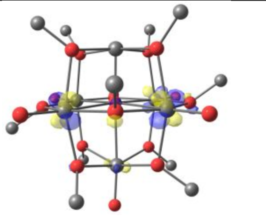	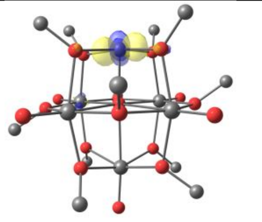	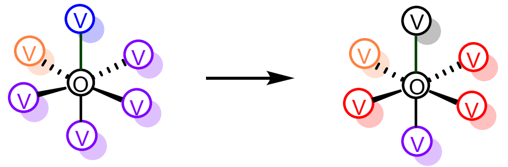 Charge-transfer-driven redistribution
λ = 405.3 nm	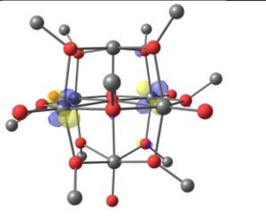	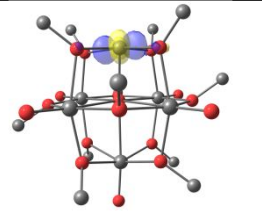	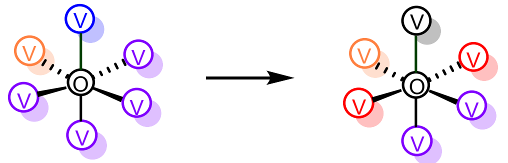 Charge-transfer-driven redistribution
λ = 394.5 nm	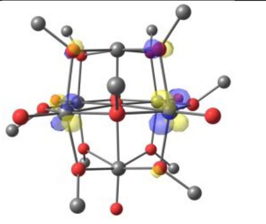	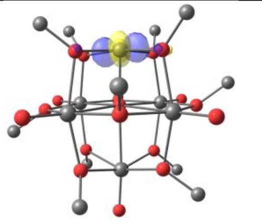	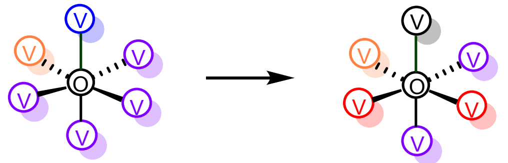 Charge-transfer-driven redistribution
λ = 393.7 nm	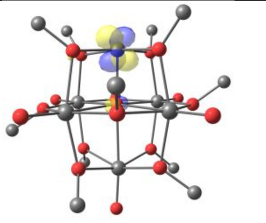	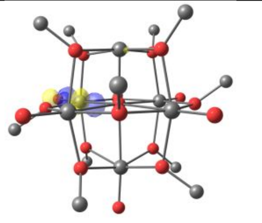	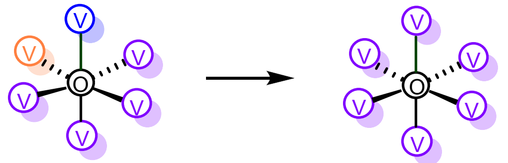 Charge transfers V(III) → V(V)

Table S2. NTO for the IVCT of compound ***2-trans***. Color scheme: V(III) in blue, V(IV) in purple, V(V) in orange, V(II) in black, and delocalized vanadium centers in red.

Excitation	HOTO	LUTO	Transition presentation
λ = 823.0 nm	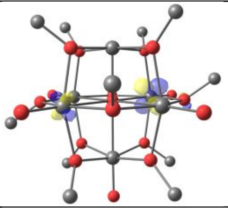	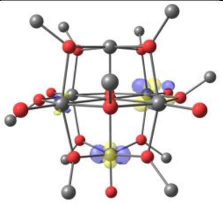	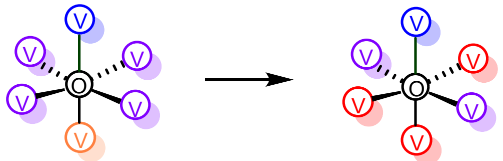 Delocalization-driven redistribution
λ = 762.9 nm	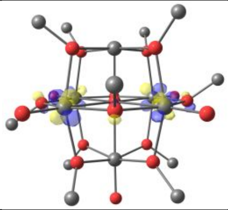	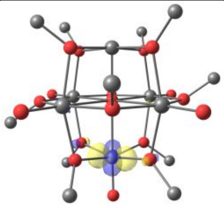	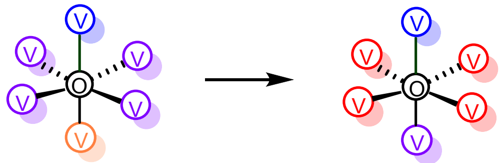 Charge-transfer-driven redistribution
λ = 693.8 nm	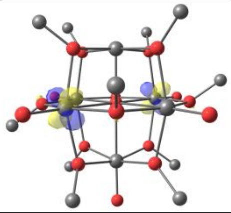	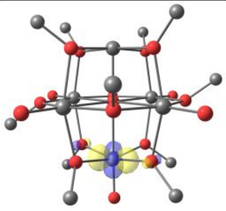	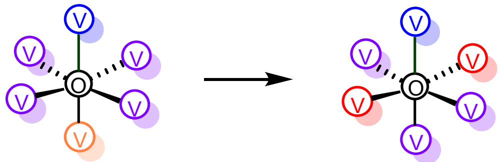 Charge-transfer-driven redistribution
λ = 678.3 nm	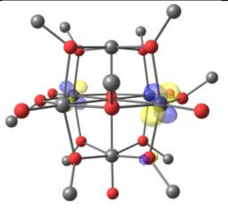	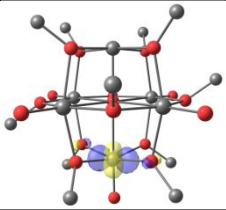	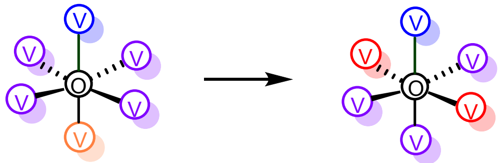 Charge-transfer-driven redistribution
λ = 642.5 nm	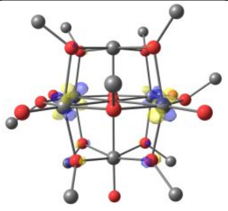	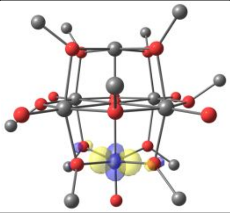	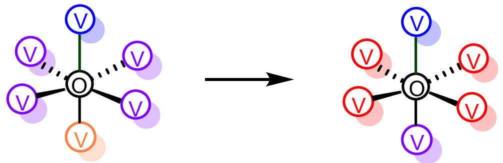 Charge-transfer-driven redistribution
λ = 541.6 nm	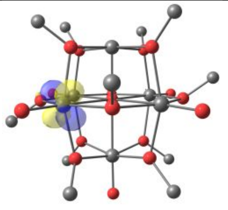	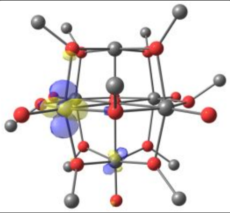	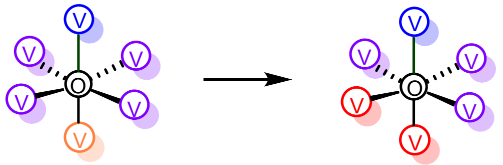 Delocalization-driven redistribution
λ = 504.0 nm	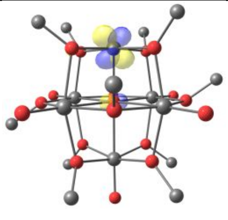	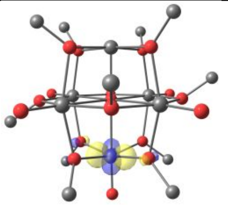	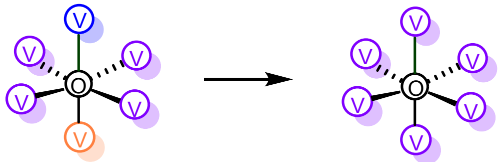 Charge transfers V(III) → V(V)

Table S2 (continue). NTO for IVCT of compound ***2-trans***

λ = 501.7 nm	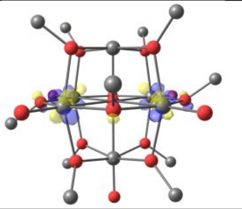	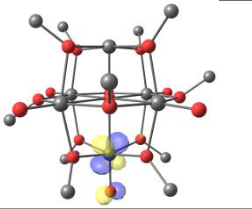	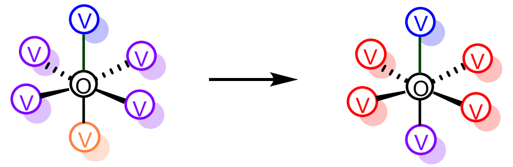 Charge-transfer-driven redistribution
λ = 462.3 nm	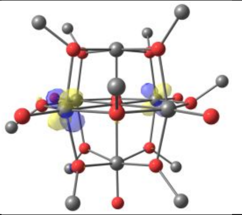	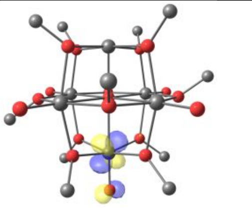	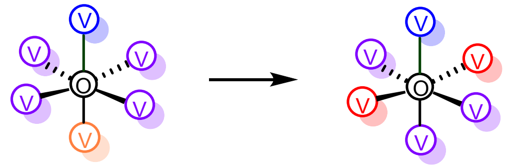 Charge-transfer-driven redistribution
λ = 425.3 nm	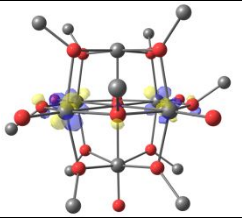	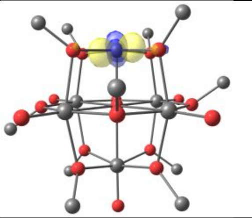	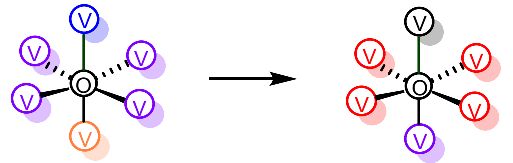 Charge-transfer-driven redistribution
λ = 404.8 nm	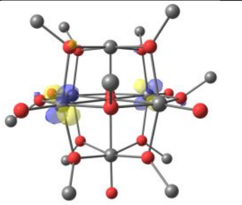	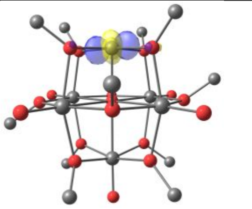	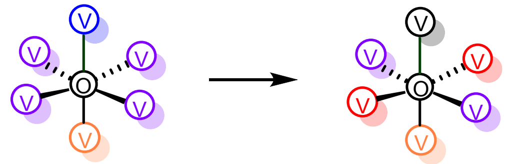 Charge-transfer-driven redistribution
λ = 402.6 nm	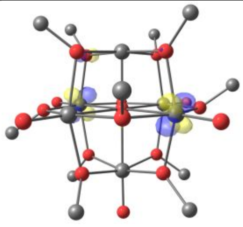	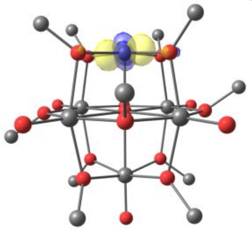	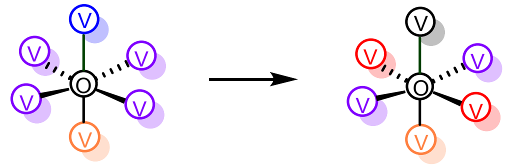 Charge-transfer-driven redistribution
λ = 387.8 nm	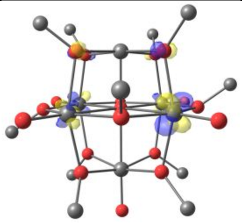	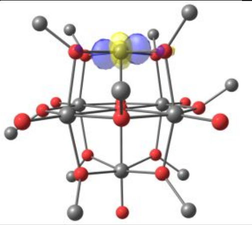	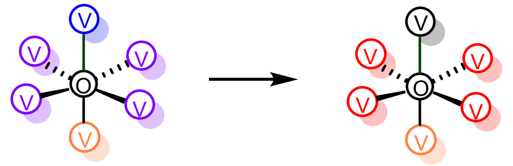 Charge-transfer-driven redistribution

Table S3. NTO for the IVCT of compound **2-MeO-*AII-IV***. Color scheme: V(III) in blue, V(IV) in purple, V(V) in orange, and delocalized vanadium centers in red.

Excitation	HOTO	LUTO	Transition presentation
λ = 667.5 nm	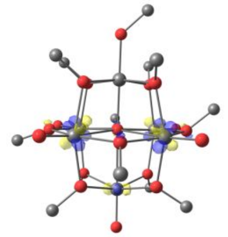	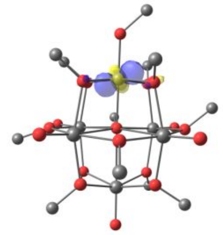	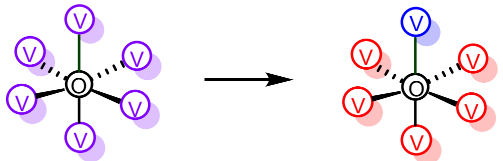 Charge-transfer-driven redistribution
λ = 587.7 nm	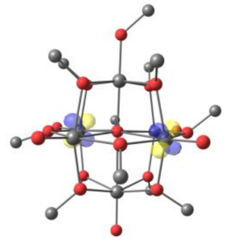	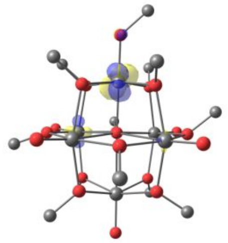	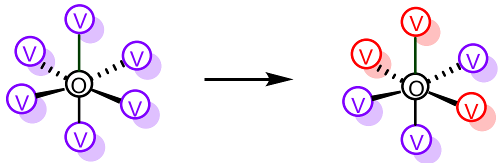 Delocalization-driven redistribution
λ = 581.1 nm	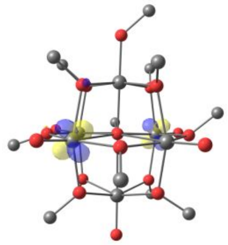	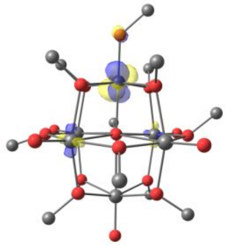	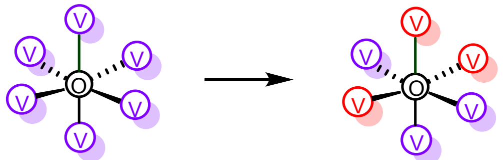 Delocalization-driven redistribution
λ = 562.0 nm	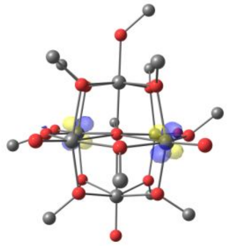	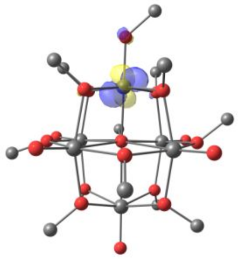	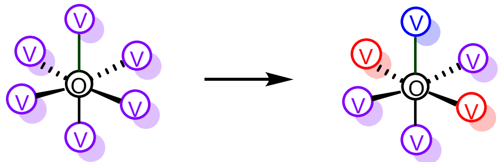 Charge-transfer-driven redistribution
λ = 551.1 nm	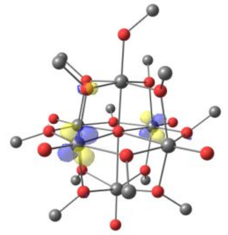	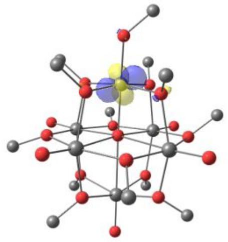	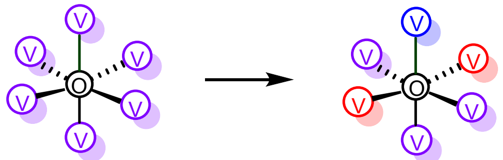 Charge-transfer-driven redistribution
λ = 531.1 nm	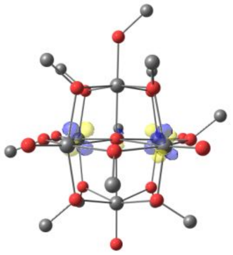	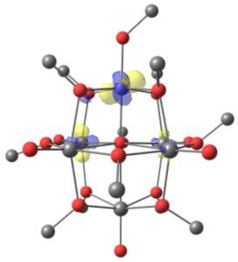	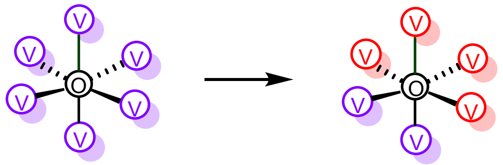 Delocalization-driven redistribution

Table S3 (continue). NTO for **MeO-2-AII(V)**

λ = 516.2 nm	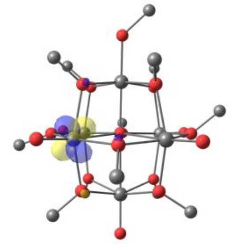	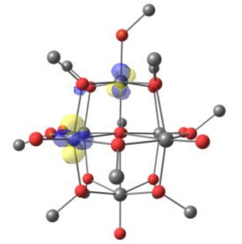	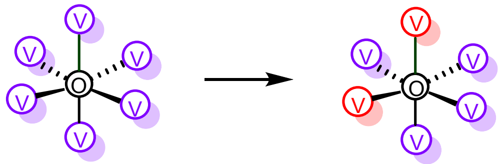 Delocalization-driven redistribution
λ = 499.8 nm	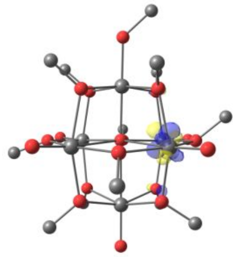	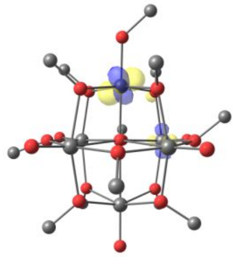	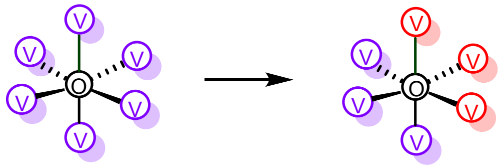 Delocalization-driven redistribution
λ = 472.6 nm	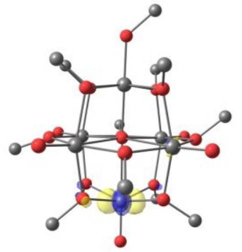	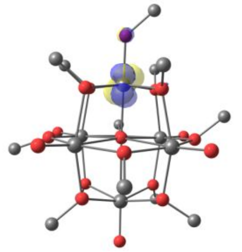	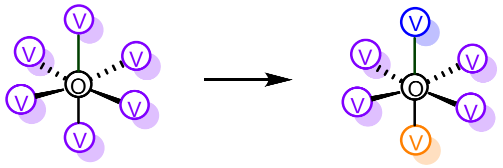 V(IV) to V(IV) IVCT

Table S4. Distances in Angstroms between the central μ_6-oxo and the vanadium centers of species with no ligand.

		Species		
**Bond**	**Position**	**2-*cis***	**2-*all-IV***	**2-*trans***
**V**	Axial	2.01	1.79	1.98
**V=O**	Equatorial	2.35-2.36	2.34-2.43	2.23-2.43
**V=O**	Axial	2.25	2.49	2.38

Table S5. Energies in kcal/mol relative to the ground state for the ***2-trans*** geometry with different ligands at CASSCF and CASPT2 level of theory.

State	Total Spin	CASSCF	CASPT2
Septet	3	0.1	0.6
Quintet	2	0.0	0.5
Triplet	1	0.0	0.0
Singlet	0	0.1	0.2

Table S6. Energies in kcal/mol relative to the ground state for the ***2-all-IV*** geometry with different ligands at CASPT2 level of theory.

State	Total Spin	CASSCF	CASPT2
Septet	3	0.4	1.2
Quintet	2	0.1	0.4
Triplet	1	0.0	0.1
Singlet	0	0.0	0.0

Table S7. Energies in kcal/mol relative to the ground state for the ***2-cis*** geometry with different ligands at CASPT2 level of theory.

State	Total Spin	CASSCF	CASPT2
Septet	3	0.1	1.6
Quintet	2	0.0	0.5
Triplet	1	0.0	0.0
Singlet	0	0.0	0.9

Table S8. Energies in kcal/mol relative to the lowest state for compound **2** at CASSCF level of theory.

State	Total Spin	2-*trans*	2-*all-IV*	2-*cis*
Septet	3	0.1	14.9	6.0
Quintet	2	0.0	14.7	5.9
Triplet	1	0.0	14.6	5.9
Singlet	0	0.1	14.5	5.9

Table S9. Energies in kcal/mol relative to the lowest state compound **2** ligand at CASPT2 level of theory.

**State**	**Total Spin**	**2-*trans***	**2-*all-IV***	**2-*cis***
Septet	3	0.6	30.8	6.1
Quintet	2	0.5	30.0	5.0
Triplet	1	0.0	29.7	4.5
Singlet	0	0.2	29.6	5.5

Table S10. LoProp charges and Mulliken spin density of vanadium centers the septet states for **2-*cis***, **2-*all-IV***, and **2-*trans*** compounds.

		**LoProp charges**			**Spin density**		
**State**	**Position**	**2-*trans***	**2-*all-IV***	**2-*cis***	**2-*trans***	**2-*all-IV***	**2-*cis***
**V**	Axial	1.78	1.96	1.77	1.96	0.96	1.96
**V=O**	Equatorial	1.75	1.74	1.75	0.98	0.98	0.98
**V=O**	Equatorial	1.76	1.76	1.91	0.98	0.98	0.00
**V=O**	Equatorial	1.75	1.74	1.76	0.98	0.98	0.98
**V=O**	Equatorial	1.76	1.76	1.76	0.98	0.98	0.98
**V=O**	Axial	1.91	1.75	1.75	0.00	0.98	0.98

Table S11. LoProp charges and Mulliken spin density of vanadium centers the quintet states for species **2-*cis***, **2-*all-IV***, and **2-*trans*** compounds.

		LoProp charges			Spin density		
**State**	**Position**	**2-*trans***	**2-*all-IV***	**2-*cis***	**2-*trans***	**2-*all-IV***	**2-*cis***
**V**	Axial	1.78	1.96	1.77	1.96	0.95	1.95
**V=O**	Equatorial	1.75	1.74	1.75	0.48	0.97	0.94
**V=O**	Equatorial	1.76	1.76	1.91	0.10	0.82	0.00
**V=O**	Equatorial	1.75	1.74	1.76	0.56	0.97	0.79
**V=O**	Equatorial	1.76	1.76	1.76	0.81	0.61	0.75
**V=O**	Axial	1.91	1.75	1.75	0.00	-0.44	-0.52

Table S12. LoProp charges and Mulliken spin density of vanadium centers the triplet states for 2-cis, 2-all-IV, and 2-trans compounds.

		**LoProp charges**			**Spin density**		
**State**	**Position**	**2-*trans***	**2-*all-IV***	**2-*cis***	**2-*trans***	**2-*all-IV***	**2-*cis***
**V**	Axial	1.78	1.96	1.77	1.95	0.83	1.94
**V=O**	Equatorial	1.75	1.74	1.75	-0.08	0.70	-0.03
**V=O**	Equatorial	1.76	1.76	1.91	0.04	0.07	0.00
**V=O**	Equatorial	1.75	1.74	1.76	-0.09	0.36	0.14
**V=O**	Equatorial	1.76	1.76	1.75	0.14	0.07	0.06
**V=O**	Axial	1.91	1.75	1.75	0.00	-0.10	-0.14

Table S13. LoProp charges of vanadium centers the singlet states for species **2-*cis***, **2-*all-IV***, and **2-*trans***.

		LoProp charges		
**State**	**Position**	**2-*trans***	**2-*all-IV***	**2-*cis***
**V**	Axial	1.78	1.96	1.77
**V=O**	Equatorial	1.75	1.74	1.75
**V=O**	Equatorial	1.76	1.76	1.91
**V=O**	Equatorial	1.75	1.74	1.76
**V=O**	Equatorial	1.76	1.76	1.75
**V=O**	Axial	1.91	1.75	1.75

Table S14. Hirshfeld Fragmentation Analysis of Hole P_h and Electron P_e for 5 →1 CTDR excitation at λ = 667.5 nm in the **2-MeO-*all-IV*** topology. Color code: V(IV) in purple, V(III) in blue, and delocalized centers in red.

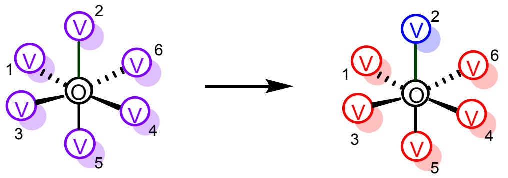

Atom	P_h (%)	P_e (%)	ΔP (%) = P_h - P_e
V^1	16.3	0.9	-15.4
V^2	1.9	70.5	68.6
V^3	12.2	0.8	-11.4
V^4	12.9	0.8	-12.1
V^5	9.7	0.1	-9.6
V^6	12.1	0.8	-11.3

Table S15. Hirshfeld Fragmentation Analysis of Hole P_h and Electron P_e for 4 → 1 CTDR excitation at λ = 642.5 nm in the **2-*trans*** topology. Color code: V(V) in orange, V(IV) in purple, V(III) in blue, delocalized centers in red.

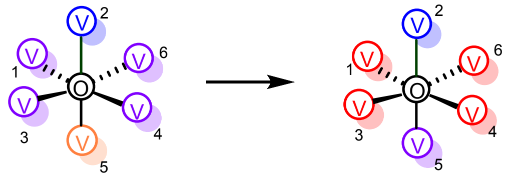

Atom	P_h (%)	P_e (%)	ΔP (%) = P_h - P_e
V^1	9.7	0.8	-11.9
V^2	1.6	0.3	-1.0
V^3	22.8	1.3	-7.6
V^4	8.8	0.8	-13.0
V^5	1.1	67.9	44.6
V^6	17.2	1.1	-3.9

Table S16. Hirshfeld Fragmentation Analysis of Hole P_h and Electron P_e for 2 →1 CTDR excitation at λ = 562.0 nm in the **2-MeO-*all-IV*** topology. Color code: V(IV) in purple, V(III) in blue, delocalized centers in red.

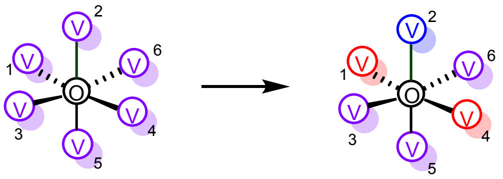

Atom	P_h (%)	P_e (%)	ΔP (%) = P_h - P_e
V^1	24.0	0.8	-23.2
V^2	0.5	69.3	-58.8
V^3	2.1	1.6	-0.5
V^4	37.6	0.8	-36.8
V^5	1.0	0.2	-0.8
V^6	0.5	0.9	0.4

Table S17. Hirshfeld Fragmentation Analysis of Hole P_h and Electron P_e for 2-sites 1 →1 IVCT excitation at λ = 504.0 nm in the ***2-trans*** topology. Color code: V(V) in orange, V(IV) in purple, and V(III) in blue.

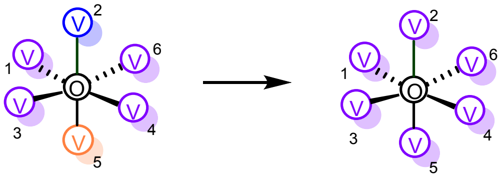

Atom	P_h (%)	P_e (%)	ΔP (%) = P_h - P_e
V^1	2.2	0.8	-1.4
V^2	65.8	4.4	-61.4
V^3	0.9	1.3	0.4
V^4	2.3	1.4	-0.9
V^5	0.5	64.6	64.1
V^6	0.6	1.1	0.5

Table S18. Hirshfeld Fragmentation Analysis of Hole P_h and Electron P_e for 4-sites DDR excitation at λ = 531.1 nm in the **2-MeO-*all-IV*** topology. Color code: V(IV) in purple and delocalized centers in red.

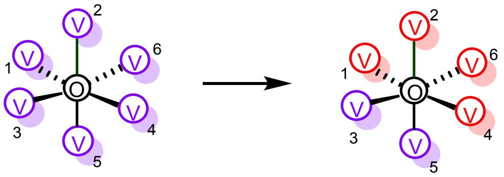

Atom	P_h (%)	P_e (%)	ΔP (%) = P_h - P_e
V^1	15.4	10.1	-5.3
V^2	0.6	30.1	29.5
V^3	5.8	4.0	-1.8
V^4	17.2	15.0	-2.2
V^5	9.4	7.9	-1.5
V^6	16.7	4.9	-11.8

Table S19. Hirshfeld Fragmentation Analysis of Hole P_h and Electron P_e for 3-sites DDR excitation at λ = 823.0 nm in the ***2-trans*** topology. Color code: V(V) in orange, V(IV) in purple, V(III) in blue, delocalized centers in red.

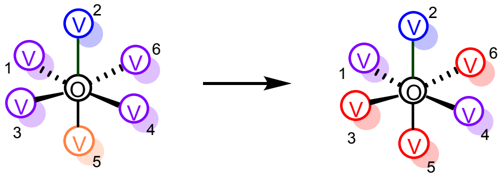

Atom	P_h (%)	P_e (%)	ΔP (%) = P_h - P_e
V^1	21.0	17.2	-5.3
V^2	1.7	0.9	-0.8
V^3	8.6	5.3	-3.3
V^4	27.9	24.5	-3.4
V^5	1.5	21.1	19.6
V^6	3.0	1.0	-2.0

Table S20. Hirshfeld Fragmentation Analysis of Hole P_h and Electron P_e for 2-sites DDR excitation at λ = 516.2 nm in the **2-MeO-*AII-IV*** topology. Color V(IV) in purple and delocalized centers in red.

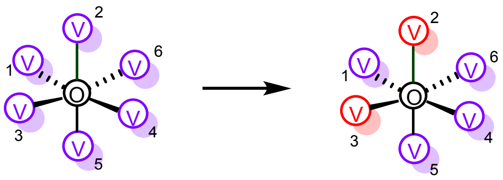

Atom	P_h (%)	P_e (%)	ΔP (%) = P_h - P_e
V^1	7.6	6.5	-1.1
V^2	0.9	35.9	35.0
V^3	25.7	15.1	-10.6
V^4	18.7	8.1	-10.6
V^5	1.8	2.5	0.7
V^6	9.8	3.2	-6.6

References

(1) Neese, F. The ORCA program system. *WIREs Computational Molecular Science* **2012**, *2* (1), 73-78. DOI: https://doi.org/10.1002/wcms.81.

(2) Neese, F. Software Update: The ORCA Program System—Version 6.0. *WIREs Computational Molecular Science* **2025**, *15* (2), e70019. DOI: https://doi.org/10.1002/wcms.70019.

(3) Adamo, C.; Barone, V. Toward reliable density functional methods without adjustable parameters: The PBE0 model. *The Journal of Chemical Physics* **1999**, *110* (13), 6158-6170. DOI: 10.1063/1.478522 (acccessed 12/17/2025).

(4) Ernzerhof, M.; Scuseria, G. E. Assessment of the Perdew–Burke–Ernzerhof exchange-correlation functional. *The Journal of Chemical Physics* **1999**, *110* (11), 5029-5036. DOI: 10.1063/1.478401 (acccessed 12/17/2025).

(5) Weigend, F.; Ahlrichs, R. Balanced basis sets of split valence, triple zeta valence and quadruple zeta valence quality for H to Rn: Design and assessment of accuracy. *Physical Chemistry Chemical Physics* **2005**, *7* (18), 3297-3305, 10.1039/B508541A. DOI: 10.1039/B508541A.

(6) Caldeweyher, E.; Mewes, J.-M.; Ehlert, S.; Grimme, S. Extension and evaluation of the D4 London-dispersion model for periodic systems. *Physical Chemistry Chemical Physics* **2020**, *22* (16), 8499-8512, 10.1039/D0CP00502A. DOI: 10.1039/D0CP00502A.

(7) Marenich, A. V.; Cramer, C. J.; Truhlar, D. G. Universal Solvation Model Based on Solute Electron Density and on a Continuum Model of the Solvent Defined by the Bulk Dielectric Constant and Atomic Surface Tensions. *The Journal of Physical Chemistry B* **2009**, *113* (18), 6378-6396. DOI: 10.1021/jp810292n.

(8) Ravelli, D.; Dondi, D.; Fagnoni, M.; Albini, A.; Bagno, A. Predicting the UV spectrum of polyoxometalates by TD-DFT. *Journal of Computational Chemistry* **2011**, *32* (14), 2983-2987. DOI: https://doi.org/10.1002/jcc.21879.

(9) López, X.; Carbó, J. J.; Bo, C.; Poblet, J. M. Structure, properties and reactivity of polyoxometalates: a theoretical perspective. *Chemical Society Reviews* **2012**, *41* (22), 7537-7571, 10.1039/C2CS35168D. DOI: 10.1039/C2CS35168D.

(10) Petel, B. E.; Brennessel, W. W.; Matson, E. M. Oxygen-Atom Vacancy Formation at Polyoxovanadate Clusters: Homogeneous Models for Reducible Metal Oxides. *Journal of the American Chemical Society* **2018**, *140* (27), 8424-8428. DOI: 10.1021/jacs.8b05298.

(11) Andersson, K.; Malmqvist, P. Å.; Roos, B. O. Second-order perturbation theory with a complete active space self-consistent field reference function. *The Journal of Chemical Physics* **1992**, *96* (2), 1218-1226. DOI: 10.1063/1.462209 (acccessed 1/8/2026).

(12) Andersson, K.; Malmqvist, P. A.; Roos, B. O.; Sadlej, A. J.; Wolinski, K. Second-order perturbation theory with a CASSCF reference function. *The Journal of Physical Chemistry* **1990**, *94* (14), 5483-5488. DOI: 10.1021/j100377a012.

(13) Li Manni, G.; Fdez. Galván, I.; Alavi, A.; Aleotti, F.; Aquilante, F.; Autschbach, J.; Avagliano, D.; Baiardi, A.; Bao, J. J.; Battaglia, S.; et al. The OpenMolcas Web: A Community-Driven Approach to Advancing Computational Chemistry. *Journal of Chemical Theory and Computation* **2023**, *19* (20), 6933-6991. DOI: 10.1021/acs.jctc.3c00182.

(14) Forsberg, N.; Malmqvist, P.-Å. Multiconfiguration perturbation theory with imaginary level shift. *Chemical Physics Letters* **1997**, *274* (1), 196-204. DOI: https://doi.org/10.1016/S0009-2614(97)00669-6.
(15) Ghigo, G.; Roos, B. O.; Malmqvist, P.-Å. A modified definition of the zeroth-order Hamiltonian in multiconfigurational perturbation theory (CASPT2). *Chemical Physics Letters* **2004**, *396* (1), 142-149. DOI: https://doi.org/10.1016/j.cplett.2004.08.032.
(16) Douglas, M.; Kroll, N. M. Quantum electrodynamical corrections to the fine structure of helium. *Annals of Physics* **1974**, *82* (1), 89-155. DOI: https://doi.org/10.1016/0003-4916(74)90333-9.
(17) Hess, B. A. Relativistic electronic-structure calculations employing a two-component no-pair formalism with external-field projection operators. *Physical Review A* **1986**, *33* (6), 3742-3748. DOI: 10.1103/PhysRevA.33.3742.
(18) Roos, B. O.; Veryazov, V.; Widmark, P.-O. Relativistic atomic natural orbital type basis sets for the alkaline and alkaline-earth atoms applied to the ground-state potentials for the corresponding dimers. *Theoretical Chemistry Accounts* **2004**, *111* (2), 345-351. DOI: 10.1007/s00214-003-0537-0.
(19) Roos, B. O.; Lindh, R.; Malmqvist, P.-Å.; Veryazov, V.; Widmark, P.-O. New Relativistic ANO Basis Sets for Transition Metal Atoms. *The Journal of Physical Chemistry A* **2005**, *109* (29), 6575-6579. DOI: 10.1021/jp0581126.
(20) Aquilante, F.; Autschbach, J.; Baiardi, A.; Battaglia, S.; Borin, V. A.; Chibotaru, L. F.; Conti, I.; De Vico, L.; Delcey, M.; Fdez. Galván, I.; et al. Modern quantum chemistry with [Open]Molcas. *The Journal of Chemical Physics* **2020**, *152* (21). DOI: 10.1063/5.0004835 (acccessed 1/8/2026).
(21) Martin, R. L. Natural transition orbitals. *The Journal of Chemical Physics* **2003**, *118* (11), 4775-4777. DOI: 10.1063/1.1558471 (acccessed 3/9/2026).
(22) Hirshfeld, F. L. Bonded-atom fragments for describing molecular charge densities. *Theoretica chimica acta* **1977**, *44* (2), 129-138. DOI: 10.1007/BF00549096.
(23) Heidar-Zadeh, F.; Ayers, P. W.; Verstraelen, T.; Vinogradov, I.; Vöhringer-Martinez, E.; Bultinck, P. Information-Theoretic Approaches to Atoms-in-Molecules: Hirshfeld Family of Partitioning Schemes. *The Journal of Physical Chemistry A* **2018**, *122* (17), 4219-4245. DOI: 10.1021/acs.jpca.7b08966.
(24) Lu, T. A comprehensive electron wavefunction analysis toolbox for chemists, Multiwfn. *The Journal of Chemical Physics* **2024**, *161* (8). DOI: 10.1063/5.0216272 (acccessed 3/9/2026).
(25) Lu, T.; Chen, F. Multiwfn: A multifunctional wavefunction analyzer. *Journal of Computational Chemistry* **2012**, *33* (5), 580-592. DOI: https://doi.org/10.1002/jcc.22885.
